# Molten Salt‐Assisted Synthesis of Porous Precious Metal‐Based Single‐Atom Catalysts for Oxygen Reduction Reaction

**DOI:** 10.1002/advs.202410784

**Published:** 2024-12-31

**Authors:** Chenming Fan, Xin Gao, Pengyi Tang, Qiang Wang, Bing Li

**Affiliations:** ^1^ School of Mechanical and Power Engineering East China University of Science and Technology Shanghai 200237 P. R. China; ^2^ 2020 X‐Lab Shanghai Institute of Microsystem and Information Technology Chinese Academy of Sciences Shanghai 200050 P. R. China; ^3^ National Key Laboratory of Materials for Integrated Circuits Shanghai Institute of Microsystem and Information Technology Chinese Academy of Sciences Shanghai 200050 P. R. China

**Keywords:** atomically dispersed precious metal, molten salt‐assisted pyrolysis, oxygen reduction reaction, porous nitrogen‐doped carbon

## Abstract

Precious metal‐based single‐atom catalysts (PM‐SACs) hosted in N‐doped carbon supports have shown new opportunities to revolutionize cathodic oxygen reduction reaction (ORR). However, stabilizing the high density of PM‐N_x_ sites remains a challenge, primarily due to the inherently high free energy of isolated metal atoms, predisposing them to facile atomic agglomeration. Herein, a molten salt‐assisted synthesis strategy is proposed to prepare porous PM_1_/N‐C_Pores_ (PM = Ru, Pt, and Pd) electrocatalysts with densely accessible PM‐N_x_ sites. A hierarchically porous N‐doped carbon substrate (N‐C_Pores_), synthesized via the NaCl‐assisted pyrolysis of zeolitic imidazolate framework‐8, effectively improves the utilization of PM‐N_x_ sites by increased reactants accessible surface area and reduced mass transfer resistance. In accordance with theoretical calculations, the as‐prepared Ru_1_/N‐C_Pores_, featuring superior intrinsic active Ru‐N_4_ sites, exhibit outstanding ORR turnover frequency of 6.19 e^−^ site^−1^ s^−1^, and outperforms the commercial Pt/C with a 5.3‐fold of mass activity (5.83 ± 0.61 A mg^−1^) at 0.8 V versus reversible hydrogen electrode. The commendable activity and stability of Ru_1_/N‐C_Pores_ in a real fuel cell device further affirm its practical applicability.

## Graphical abstract

A series of porous precious metal‐based single‐atom electrocatalysts (PM_1_/N‐C_Pores_, PM = Ru, and Pt, Pd), fabricated by molten salt‐assisted pyrolysis strategy, achieved a high density of catalytically accessible PM single‐atom sites. Owing to the hierarchical porous structure and the abundant highly active Ru single‐atom sites, the as‐prepared Ru_1_/N‐C_Pores_ exhibit significantly promoted oxygen reduction reaction (ORR) activity in acid electrolytes.

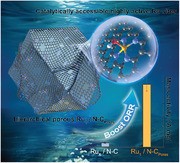

## 1 Introduction

The sluggish four‐electron oxygen reduction reaction (ORR) at the cathode in proton exchange membrane fuel cells (PEMFCs) typically necessitate a considerable amount of Pt‐based electrocatalysts; nevertheless, the high price of precious metal (PM) has severely hampered the commercialization of PEMFCs.^[^ ^1^, ^2^, ^3^ ^]^ Hitherto, the benchmark ORR catalysts exist mainly in the form of metallic Pt‐based nanoparticles, with only a few exposed surface Pt atoms participating directly in the reaction. In this context, single‐atom catalysts (SACs), theoretically capable of providing 100% atom utilization, have emerged as a highly promising category that can provide ample adsorption sites for oxygen‐containing intermediates.^[^ ^4^, ^5^, ^6^ ^]^ Recent advancements have highlighted a series of SACs, featuring atomically dispersed metals anchored on N‐doped carbon (M‐N‐C).^[^ ^7^, ^8^, ^9^ ^]^ Within these SACs boasting an abundance of metal‐N_x_ (M‐N_x_) moieties as active sites, transition metal centers, encompassing elements such as Fe, Co, Ni, Mn, Cu, etc., showcasing promising ORR activity.^[^ ^10^, ^11^, ^12^, ^13^, ^14^ ^]^ It is worth mentioning that participation in the Fenton reaction significantly reduces the activity of transition metal‐based SACs, particularly Fe–N–C SACs, as opposed to M‐N‐C SACs based on PM‐N_x_ (PM = Ru, Rh, Pd, Ir, Pt, etc.) cites.^[^ ^15^, ^16^, ^17^, ^18^ ^]^ Ru and Ir metals with stable outer shell electron arrangements, whose ions have been shown to weakly interact with hydrogen peroxide via Fenton reaction, thus offering improved resistance to deactivation over extended periods.^[^ ^19^, ^20^ ^]^

Typically, pyrolysis of precursors containing metal, carbon, and nitrogen is used to prepare M‐N‐C SACs, which frequently results in low‐density dispersion of the M‐N_x_ sites.^[^ ^21^ ^]^ Micropores with short pore wall length facilitate the formation of M‐N_x_ sites by bridging the two‐pore walls under high temperature, while surface micropore‐hosted M‐N_x_ sites are well suited for ORR due to their easily accessible to reactants.^[^ ^22^ ^]^ By increasing the catalytic accessible surface area and reducing the mass transfer resistance that can arise from relying solely on micropores, the incorporation of mesopores/macropores can further enhance the utilization of M‐N_x_ sites.^[^ ^23^, ^24^, ^25^ ^]^ Therefore, a well‐designed hierarchical porous structure is crucial for obtaining a high density of reactants accessible active sites at low metal loading, thereby achieving ORR performance comparable to that of Pt/C.

Zeolitic imidazolate framework‐8 (ZIF‐8) crystals, regarded as most widely used sacrificial template for preparing microporous N‐doped carbon, are capable of forming uniform metal‐nitrogen coordinated environment by partially replacing Zn ions with doped metal ions.^[^ ^8^, ^26^, ^27^ ^]^ Unlike Fe, Co, and Ni ions, the encapsulation of PM ions in the ZIF‐8 structure is considerably hampered by the pronounced disparities in both size and properties relative to Zn ions, resulting in merely 0.2–0.5 wt.% PM loading on N‐doped carbon supports post‐pyrolysis.^[^ ^28^, ^29^ ^]^ Simultaneously, the micropores shrinkage and collapse during the pyrolysis of PM‐impregnated ZIF‐8 precursors facilitates the manifestation of the active sites predominantly in nanoparticulate form, in turn, further precipitates a diminution in the overall availability of the PM‐N_x_ sites.^[^ ^30^, ^31^, ^32^ ^]^ Both the soft and hard template methods have proven to be effective for the synthesis of ZIF‐8 derived porous N‐doped carbon.^[^ ^33^, ^34^ ^]^ However, the soft template method requires specific interactions between the precursor and template, whereas the hard template method typically involves complex post‐pyrolysis etching process. When synthesizing M‐N‐C SACs in an open atmosphere, volatilization of metals and nitrogen may lead to the loss of active substances, which reduces the density of active sites. Since the molten salt acts as an invisible reactor during the reaction process, it is feasible to form porous structure by shielding the loss of active material through the molten salt phase transition.^[^ ^35^ ^]^ Consequently, researchers have endeavored to incorporate NaCl as a surface textural modifier during the pyrolysis process to construct N‐doped carbon with porous structure.^[^ ^36^, ^37^, ^38^ ^]^ In prior investigations employing NaCl, the salt infiltrated the entire structure of the ZIF‐8 crystal, causing the morphology and microporosity of the ZIF‐8 crystal to deteriorate due to delamination, yielding N‐doped carbon nanosheets with diminished porosity. Recently, Wang adopted a NaCl‐assisted pyrolysis strategy to maintain the micropore richness of the ZIF‐8 precursor.^[^ ^39^ ^]^ By post‐loading Fe single atoms on hierarchically porous N‐doped carbon supports, the synthesized Fe–N–C catalysts exhibit an abundance of Fe‐N_x_ sites for ORR, providing a novel perspective on the synthesis of SACs. This inspires further research aimed at investigating the role of hierarchical porous N‐doped carbon in the construction and stabilization of PM‐N_x_ sites.

Herein, we employ a molten salt‐assisted pyrolysis strategy to fabricate porous N‐doped carbon supports (N‐C_Pores_), which are then utilized to immobilize PM single atoms, synthesizing a series of PM_1_/N‐C_Pores_ (PM = Ru, Pt, and Pd) for ORR catalysis. Contrasted with traditional encapsulation pyrolysis synthesis method, the hierarchical porous N‐doped carbon supports with high specific surface areas, obtained through NaCl‐assisted pyrolysis of ZIF‐8, are capable of anchoring densely accessible high active PM‐N_x_ sites. Through rotating ring disk electrode (RRDE) tests, it is confirmed that a series of PM_1_/N‐C_Pores_ exhibit enhanced ORR activity when compared with PM clusters (PM_x_/N‐C) achieved by traditional encapsulation pyrolysis strategy. Remarkably, the as‐prepared Ru_1_/N‐C_Pores_ shows outstanding ORR activity and stability in both RRDE and PEMFCs tests. The atomic Ru‐N_4_ active sites in Ru_1_/N‐C_Pores_ have been examined and confirmed by aberration‐corrected high‐angle annular dark‐field scanning transmission electron microscopy (HAADF‐STEM) and X‐ray absorption spectroscopy (XAS). In conjunction with density functional theory (DFT) calculations, mechanistic explanations for the excellent activity of Ru_1_/N‐C_Pores_ are offered, focusing on the coordination environment and electronic structure of the active centers.

## 2 Results and Discussion

### 2.1 Synthesis and Characterization of PM_1_/N‐C_Pores_

**Figure 1:**
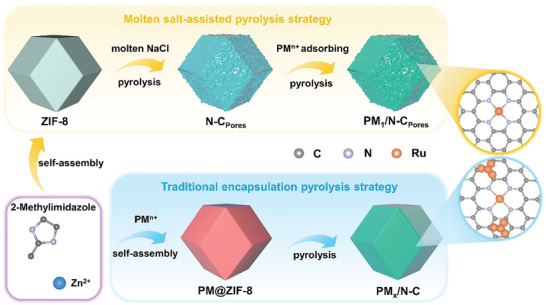
Schematic illustration of molten salt‐assisted pyrolysis and traditional encapsulation pyrolysis strategy.

A series of PM‐based (PM = Ru, Pt, and Pd) SACs were prepared by molten salt‐assisted pyrolysis strategy and compared with traditional encapsulation pyrolysis strategy, as illustrated in **Figure** **1**. The synthesis process of PM_1_/N‐C_Pores_ electrocatalysts encompasses the preparation of highly porous N‐C_Pores_ support, followed by the subsequent loading of isolated PM atoms. A physical amalgamation of ZIF‐8 with NaCl at a weight ratio of 1:2 was treated at 950 °C for 1 h, under a continuous 5% H_2_/Ar purge. When heated to over 800 °C, a small amount of molten NaCl encapsulated the carbonizing ZIF‐8 without infiltrating the entire structure (Figure S1, Supporting Information). Consequently, the NaCl occupied the surface micropores and the pores left by Zn sublimation, thus forming a porous structure. The resulting hierarchical porous N‐C_Pores_ substrate was heat treated once more in a reducing environment after PM ions were adsorbed onto it. Table S1 (Supporting Information) records the relevant PM loading of PM_1_/N‐C_Pores_ (Ru: 1.01 wt.%; Pt: 0.97 wt.%; Pd: 1.12 wt.%) according to the inductively coupled plasma optical emission spectroscopy (ICP‐OES) measurements. For comparison, PM_x_/N‐C electrocatalysts (Ru: 1.34 wt.%; Pt: 1.06 wt.%; Pd: 0.97 wt.%) with the same PM content as PM_1_/N‐C_Pores_ electrocatalysts were manufactured using the traditional encapsulation pyrolysis strategy. PM(acac)_n_ was incorporated during the self‐assembly process of ZIF‐8, entrapping PM ions within the ZIF‐8 structure to form PM@ZIF‐8. PM_x_/N‐C electrocatalysts were obtained by pyrolysis of PM@ZIF‐8 under the same conditions as the preparation of PM_1_/N‐C_Pores_ electrocatalysts, except that NaCl was not added.

**Figure 2:**
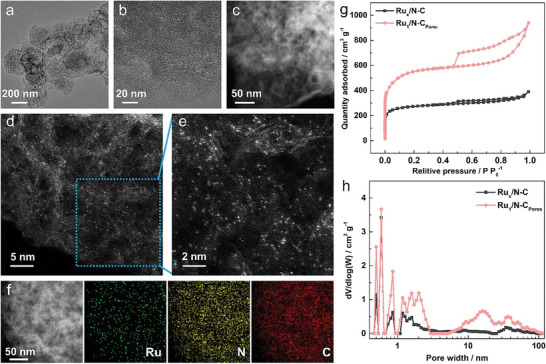
Morphology characterization of Ru_1_/N‐C_Pores_: a,b) TEM images; c) HAADF‐STEM image; d,e) Atomic‐resolution HAADF‐STEM images; f) HAADF‐STEM image and corresponding EDS elemental mapping; g,h) N_2_ adsorption/desorption isotherms and the corresponding pore size distribution curves of Ru_1_/N‐C_Pores_ and Ru_x_/N─C.

Using Ru as an illustration, by comparing the X‐ray diffraction (XRD) pattern and scanning electron microscopy (SEM) image with those of ZIF‐8, it was confirmed that the prepared Ru@ZIF‐8 maintained a well‐preserved crystalline structure (Figure S2, Supporting Information). Figure S3 (Supporting Information) shows that the N─C obtained through pyrolysis without NaCl can retain the dodecahedron morphology of the ZIF‐8 crystal, whereas the achieved N‐C_Pores_ exhibit a porous structure. Through the appearance of the (002) and (100)/(101) amorphous carbon peaks in XRD patterns (Figure S4a, Supporting Information), the complete carbonization of N─C and N‐C_Pores_ was confirmed. Furthermore, this indicates that the NaCl‐assisted pyrolysis process did not significantly alter the crystalline state of the carbon substrate. Neither the XRD patterns of Ru_1_/N‐C_Pores_ nor Ru_x_/N─C (Figure S4a, Supporting Information) display diffraction peaks indicative of Ru, which suggests, to some extent, a well‐maintained dispersion of Ru within these materials. The Raman spectra of all samples, as displayed in Figure S4b (Supporting Information), exhibited distinct peaks at ≈1350 cm^−1^ (D‐band) and 1580 cm^−1^ (G‐band), attributed to the amorphous structure of the sp^3^‐bonded carbon and the vibrations of the sp^2^‐hybridized carbon atoms in the ordered graphite, respectively.^[^ ^40^, ^41^ ^]^ Thus, the higher intensity ratio of D‐band and G‐band (I_D_/I_G_) for N‐C_Pores_ (1.07), Ru_1_/N‐C_Pores_ (1.15), and Ru_x_/N─C (1.21), in comparison to N─C (1.02), indicate that the inclusion of NaCl and the anchoring of Ru both contribute to an increased defect density within the N‐doped carbon supports. The structure and morphology of Ru_1_/N‐C_Pores_ were probed with transmission electron microscopy (TEM) and aberration‐corrected high‐angle annular dark‐field scanning transmission electron microscopy (HAADF‐STEM) in **Figure** **2**a–c. When compared to the HAADF‐STEM image of the N‐C_Pores_ in Figure S3a (Supporting Information), observable Ru nanoparticles and clusters were not detected in the Ru_1_/N‐C_Pores_, indicating that the Ru atoms do not aggregate significantly (Figure 2c). The individual bright dots observed in the atomic‐resolution HAADF‐STEM images of Ru_1_/N‐C_Pores_, as illustrated in Figure 2d,e, revealed the existence of atomically dispersed Ru atoms. Additional energy‐dispersive spectrum (EDS) elemental mappings (Figure 2f) further confirm the homogeneous distribution of Ru and N within the ZIF‐8 derived N‐doped carbon substrate. However, a substantial number of tiny particles emerged in Ru_x_/N─C, which had a comparable Ru content to Ru_1_/N‐C_Pores_. Obviously, HAADF‐STEM image and the corresponding EDS elemental mappings of Ru_x_/N─C (Figure S5, Supporting Information) make it evident that Ru particles were scattered unevenly, with no Ru elements found in the region enclosed by the red dashed lines.

The characterizations mentioned above indicate that the addition of NaCl has a considerable impact on the structure and morphology of the N‐doped carbon supports obtained through pyrolysis. By modifying the mass ratio of ZIF‐8 precursor to NaCl (1:1, 1:2, 1:4), a series of N‐C_Pores_ supports, designated as N‐C_Pores_(1:1), N‐C_Pores_ and N‐C_Pores_(1:4), were synthesized to better investigate the crucial role of NaCl in the production of PM‐based SACs. Sublimation during pyrolysis causes the elimination of Zn from the ZIF‐8 precursors. As illustrated in Table S2 (Supporting Information), the Zn content in N‐C_Pores_ (0.89 wt.%) was significantly lower than that in N─C (2.77 wt.%), which suggests that the pyrolysis of ZIF‐8, assisted by NaCl, reduces the residual Zn content in the N‐doped carbon supports.^[^ ^39^ ^]^ Additionally, after adding different amounts of NaCl, the maximum residual Zn content, 2.56 wt.%, was found in the N‐C_Pores_(1:4) when the mass ratio was 1:4, indicating that adding excess NaCl can impede the removal of Zn. The high‐resolution N 1s X‐ray photoelectron spectroscopy (XPS) spectra of all N‐doped carbon supports present pyridinic N, pyrrolic N, graphitic N, and oxidized‐N (Figure S6 and Table S3, Supporting Information).^[^ ^42^, ^43^ ^]^ The N‐doped carbon supports achieved with the addition of NaCl, especially N‐C_Pores_ (32.43%, 45.05%), all exhibited higher content of pyridinic N and graphitic N, which contribute to the formation of metal‐coordinated site and electron transfer, respectively.^[^ ^44^ ^]^ The surface area of Ru_1_/N‐C_Pores_, Ru_x_/N─C, and N‐doped carbon supports were examined by N_2_ physisorption measurements, and the corresponding pore size distribution curves were achieved using the non‐local density functional theory method (Figures 2g,h and S7 and Table S4, Supporting Information). As shown in Figures 2g and S7a (Supporting Information), N─C and Ru_x_/N─C were dominated by type I isotherms with small hysteresis loop, which is typical of microporous structure, whereas all isotherms obtained by NaCl‐assisted pyrolysis were a combination of type I and type IV, with H4 hysteresis loop, indicating the coexistence of microporous and mesoporous structure.^[^ ^45^, ^46^ ^]^ NaCl‐assisted synthesized N‐doped carbon supports showed higher BET surface area (S_BET_) than N─C (1147.98 m^2^ g^−1^); nevertheless, excessive NaCl will also harm the porous structure of pyrolytic carbon materials, as the S_BET_ of N‐C_Pores_(1:4) (1714.81 m^2^ g^−1^) was smaller than that of N‐C_Pores_ (2103.99 m^2^ g^−1^) and N‐C_Pores_(1:1) (1967.58 m^2^ g^−1^) (Figure S7 and Table S4, Supporting Information). Although the pore volume was slightly damaged by the secondary heat treatment process, Ru_1_/N‐C_Pores_ loaded on N‐C_Pores_ could still maintain a high S_BET_ and pore volume (V_pores_) (2054.50 m^2^ g^−1^, 1.45 cm^3^ g^−1^), which was considerably superior to that of Ru_x_/N─C (1032.75 m^2^ g^−1^, 0.60 cm^3^ g^−1^) (Figure 2g,h; Table S4, Supporting Information). The significant increase of micropores/small‐sized mesopores (1–3 nm) and large‐sized mesopores/macropores (10–100 nm) confirmed the hierarchical porous structure of Ru_1_/N‐C_Pores_. In addition, the Ru_1_/N‐C_Pores_ exhibited a larger micropore volume (V_micro_ = 0.78 cm^3^ g^−1^) and external surface area (S_external_ = 198.70 m^2^ g^−1^) than Ru_x_/N─C (0.38 cm^3^ g^−1^, 112.71 m^2^ g^−1^). Micropores are essential for hosting a greater number of active sites, and the incorporation of mesoporous/macroporous structure can further enhance the utilization of active sites. At the same time, active sites located near the external surface are more accessible to ORR reactants, making a higher S_external_ advantageous for the formation of catalytically accessible active sites.^[^ ^43^, ^47^ ^]^ The TEM and atomic‐resolution HAADF‐STEM images (Figure S8, Supporting Information) reveal that Ru_1_/N‐C_Pores_(1:1) and Ru_1_/N‐C_Pores_(1:4), loaded on N‐C_Pores_(1:1) and N‐C_Pores_(1:4) respectively, exhibited a porous structure akin to Ru_1_/N‐C_Pores_ (Figure 2). As evidenced in Figure S8b,d (Supporting Information), the Ru atoms within Ru_1_/N‐C_Pores_(1:1) were homogeneously distributed across the N‐C_Pores_(1:1) matrix, whereas slight agglomerations of Ru were discernible in Ru_1_/N‐C_Pores_(1:4). This observation substantiates that porous N‐doped carbon supports endowed with a high surface area facilitate the presence of Ru in the form of a single atom. NaCl can function as a pore‐forming agent, promoting the development of a hierarchical porous structure in N‐C_Pores_ (containing abundant micropores), thus enabling the electrocatalysts to provide more catalytically accessible active sites.

**Figure 3:**
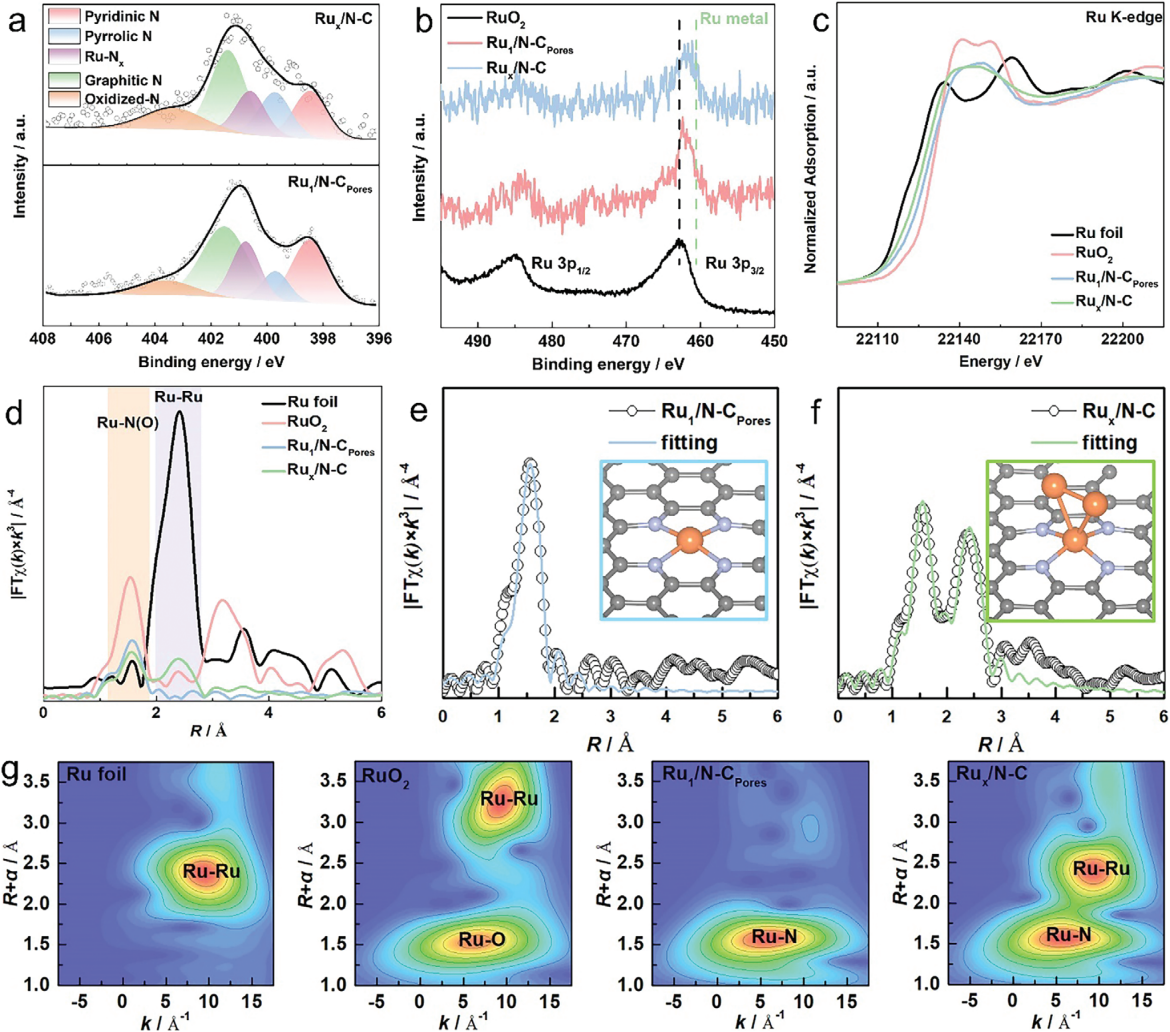
Structure and valence state analysis: a) High‐resolution N 1s XPS spectra of Ru_1_/N‐C_Pores_ and Ru_x_/N─C; b) High‐resolution Ru 3p XPS spectra of RuO_2_, Ru_1_/N‐C_Pores,_ and Ru_x_/N─C; c,d) Ru K‐edge XANES and FT‐EXAFS spectra of Ru foil, RuO_2_, Ru_1_/N‐C_Pores_ and Ru_x_/N─C; e) EXAFS fitting curve for Ru_1_/N‐C_Pores_ in *R*‐space; f) EXAFS fitting curve for Ru_x_/N─C in *R*‐space; g) WTs for the Ru K‐edge EXASF signals of Ru foil, RuO_2_, Ru_1_/N‐C_Pores_ and Ru_x_/N─C.

XPS characterization was utilized to gain insights into the chemical state of Ru_1_/N‐C_Pores_ and Ru_x_/N─C. Survey scan XPS full spectra confirm the existence of C, N, O, and Ru in all samples (Figure S9a, Supporting Information). Although detailed XPS analysis of Ru is traditionally through the detection of Ru 3d photoelectrons signal, to circumvent strong interference with the overlapped C 1s from carbon substrates (Figure S9b, Supporting Information), we adopted the Ru 3p spectra in our subsequent analyses.^[^ ^48^ ^]^ The high‐resolution N 1s spectra (**Figure** **3**a) of Ru_1_/N‐C_Pores_ and Ru_x_/N─C can be deconvoluted into five peaks, among which the newly emerged peak compared with N‐doped carbon supports (Figure S6, Supporting Information) belongs to Ru‐N_x_, indicating the existence of strong interaction between Ru center and N.^[^ ^29^ ^]^ Significantly, pyridinic N is normally regarded as potential metal‐coordinated site. Compared with Ru_x_/N─C (18.77%, 15.36%), Ru_1_/N‐C_Pores_ (25.55%, 18.61%) had higher pyridinic N and Ru‐N_x_ content, confirming that the increased microporosity facilitated by NaCl promoted the exposure of RuN_x_ active sites, thereby enhancing ORR performance. Given that in the Ru 3p spectra (Figure 3b) of both Ru_1_/N‐C_Pores_ and Ru_x_/N─C, only two weak peaks corresponding to Ru 3p_3/2_ and Ru 3p_1/2_ can be discerned, the electronic state of the Ru sites relies solely on comparing the binding energy of Ru 3p_3/2_ with that of metallic Ru (461.2 eV) and RuO_2_ (462.8 eV).^[^ ^49^ ^]^ The binding energies for the Ru 3p_3/2_ peaks in both Ru_1_/N‐C_Pores_ (462.26 eV) and Ru_x_/N─C (461.89 eV) were positioned intermediates to those observed for metallic Ru and RuO_2_, suggesting that the partial electron transfer between Ru and N placed the Ru active sites in an oxidation state. Notably, compared with Ru_x_/N─C, the Ru 3p XPS peaks of Ru_1_/N‐C_Pores_ were shifted further toward a higher binding energy direction, bringing it closer to Ru(III) and Ru(IV). The structure and coordination environment of the Ru sites in Ru_1_/N‐C_Pores_ and Ru_x_/N─C were further investigated by X‐ray absorption near edge structure (XANES) and extended X‐ray absorption fine structure (EXAFS) spectroscopies. Ru K‐edge XANES spectra (Figure 3c) with the white‐line intensity enhanced in the order Ru foil<Ru_x_/N─C<Ru_1_/N‐C_Pores_<RuO_2_ reveal the positive charge state of Ru sites in Ru_1_/N‐C_Pores_ and Ru_x_/N─C. Simultaneously, the Ru sites in Ru_1_/N‐C_Pore_ exhibited a higher oxidation state, agreeing with the XPS analysis. In the Fourier transform‐EXAFS (FT‐EXASF) spectra (Figure 3d), both Ru_1_/N‐C_Pores_ and Ru_x_/N─C had a peak ascribed to the Ru─N(O) (≈1.6 Å) scattering path, while Ru_x_/N─C also showed a peak referring to the Ru‐Ru (≈2.6 Å) scattering path. The absence of Ru─Ru scattering path confirms the atomic dispersion of Ru atoms in Ru_1_/N‐C_Pores_, in line with the morphology observation. From the least‐squares EXAFS curve‐fitting analysis (Figures 3e,f and S10 and Table S5, Supporting Information), the coordination configuration of Ru active sites in Ru_1_/N‐C_Pores_ and Ru_x_/N─C was established. The Ru─N(O) coordination number in Ru_1_/N‐C_Pores_ was estimated to be 4.0 ± 0.3, indicating that the configuration of Ru active sites was determined as Ru‐N_4_ (inset in Figure 3e). For Ru_x_/N─C, the Ru─N(O) and Ru‐Ru coordination numbers were 3.8 ± 0.6 and 2.0 ± 0.6, respectively, suggesting the presence of Ru sites in the form of Ru_3_‐N_4_ (inset in Figure 3f). Moreover, analysis of the coordination number of Ru─Ru scattering path suggests that Ru particles are present as small‐size clusters within Ru_x_/N─C, corroborating the observations made from TEM images (Figure S5, Supporting Information). Given that the magnitude of the Fourier‐transformed signal does not capture all critical details, we employed wavelet transforms (WTs) analysis, featured with simultaneous resolution in both *k* and *R* spaces, to further identify the atomic Ru dispersion (Figure 3g).^[^ ^50^ ^]^ The Ru_1_/N‐C_Pores_ showed only one intensity maximum at 5.4 Å^−1^, assigned to the Ru─N(O) contribution, whereas another intensity maximum at 9.2 Å^−1^ was detected in Ru_x_/N─C, which could be ascribed to Ru─Ru scattering path. Based on the aforementioned analysis, it is evident that the Ru_x_/N─C synthesized by traditional encapsulation pyrolysis method cannot maintain the morphology of single atoms and exists in the form of clusters. In contrast, the porous N‐C_Pores_ achieved through NaCl‐assisted ZIF‐8 pyrolysis can guarantee the well dispersion of abundant Ru‐N_4_ sites under the same metal loading.

### 2.2 ORR Electrocatalytic Performance of Catalysts

**Figure 4:**
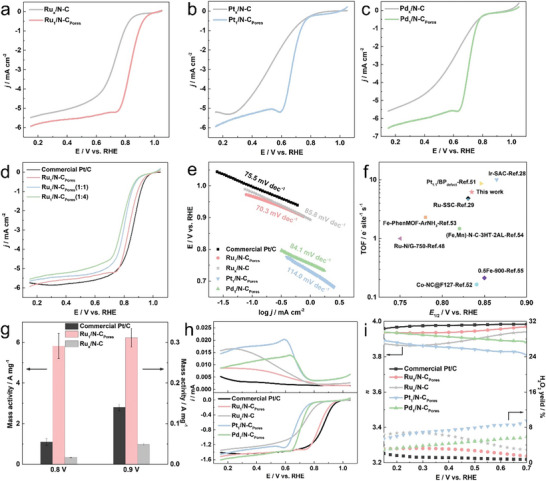
Characterization of electrocatalytic performance: a–d) ORR polarization curves recorded in O_2_‐saturated 0.1 m HClO_4_ at 1600 rpm; e) Tafel plots; f) The relationship between TOF measured at 0.8 V versus RHE and *E*_1/2_; g) Mass activities of catalysts at 0.8 and 0.9 V versus RHE; h) RRDE test recorded in O_2_‐saturated 0.1 m HClO_4_ at 1600 rpm; i) H_2_O_2_ yield and electron number transferred in ORR.

Rotating ring‐disk electrode (RRDE) was used to evaluate the ORR catalytic performance of commercial Pt/C and as‐prepared PM‐based catalysts in 0.1 m HClO_4_. The ORR polarization curves, recorded at 10 mV s^−1^, provide the onset potential (*E*_onset_) and the half‐wave potential (*E*_1/2_). All potentials were calibrated against the reversible hydrogen electrode (vs RHE). **Figure** **4**a–c compares the ORR polarization curves of PM_1_/N‐C_Pores_ and PM_x_/N‐C (PM = Ru, Pt, and Pd). A series of PM_1_/N‐C_Pores_ exhibited more positive *E*_onset_ and *E*_1/2_ relative to those of PM_x_/N‐C. Benefit from the abundant active Ru‐N_4_ sites, the ORR activity of Ru_1_/N‐C_Pores_ (*E*_onset_ = 0.980 V, *E*_1/2_ = 0.835 V) was significantly increased compared with Ru_x_/N─C (*E*_onset_ = 0.932 V, *E*_1/2_ = 0.725 V). To explore the influence of NaCl in the synthesis of PM SACs, N‐C_Pores_(1:1), N‐C_Pores_ and N‐C_Pores_(1:4) supported Ru SACs, designated as Ru_1_/N‐C_Pores_(1:1), Ru_1_/N‐C_Pores_, and Ru_1_/N‐C_Pores_(1:4) were synthesized by adjusting the mass ratio of ZIF‐8 to NaCl (1:1, 1:2, 1:4). As shown in Figure 4d, Ru_1_/N‐C_Pores_ stood out as the best ORR electrocatalyst among the series, achieving activity close to that of commercial Pt/C (*E*_onset_ = 0.982 V, *E*_1/2_ = 0.859 V), which confirmed that the optimal mass ratio of ZIF‐8 to NaCl was 1:2. According to the ORR polarization curves (Figure S11, Supporting Information) of N─C, N‐C_Pores_(1:1), N‐C_Pores_ and N‐C_Pores_(1:4), it was found that the hierarchical porous structure of N‐doped carbon supports, facilitated by NaCl, can inherently enhance the ORR activity of the catalysts to a certain extent. We further measured double‐layer capacitance (*C*_dl_) to evaluate the electrochemical surface area (ECSA) by using the CV method (Figure S12, Supporting Information). The obtained *C*_dl_ and ECSA of Ru_1_/N‐C_Pores_ were 24.18 mF cm^−2^ and 604.50 cm^2^_ECSA_, respectively, which were almost twice that of Ru_x_/N─C (14.91 mF cm^−2^ and 372.75 cm^2^_ECSA_), indicating high utilization of metal active sites. Compared with Pt_1_/N‐C_Pores_ (114.0 mV dec^−1^), Pd_1_/N‐C_Pores_ (84.1 mV dec^−1^) and commercial Pt/C (75.5 mV dec^−1^), Ru_1_/N‐C_Pores_ possessed the fastest ORR kinetics with the smallest Tafel slope of 70.3 mV dec^−1^ (Figure 4e). Moreover, Figure 4f shows the turnover frequency (TOF) of SACs to explore the intrinsic activity per active site. The TOF of Ru_1_/N‐C_Pores_ was 6.19 e^−^ site^−1^ s^−1^ (0.8 V vs RHE), which was better than other two Ru‐based SACs and was outstanding among PM‐free SACs.^[^ ^28^, ^29^, ^48^, ^51^, ^52^, ^53^, ^54^, ^55^ ^]^ The performance of Ru_1_/N‐C_Pores_ is also competitive with recently reported SACs (Table S6, Supporting Information). Considering the lower Ru loading (3 µg_Ru_ cm^−2^ vs 15 µg_Pt_ cm^−2^) on the RRDE, the mass activity (*j*_k,mass_) of Ru_1_/N‐C_Pores_ reached a 5.3‐fold (5.83 ± 0.61 A mg^−1^_Ru_ vs 1.09 ± 0.16 A mg^−1^_Pt_) and 2.2‐fold (0.31 ± 0.02 A mg^−1^_Ru_ vs 0.14 ± 0.01 A mg^−1^_Pt_) enhancement that of commercial Pt/C at 0.8 and 0.9 V versus RHE, respectively (Figure 4g; Table S7, Supporting Information).

Apart from the catalytic activity, stability is the main obstacle to the commercial application of ORR catalysts. The catalysts with optimal stability should possess idea four‐electron (4e^−^) selectivity, thereby preventing the formation of hydrogen peroxide (H_2_O_2_). To ascertain the ORR catalytic pathway of the synthesized catalysts, we conducted RRDE measurements, as depicted in Figure 4h, to monitor the yield of H_2_O_2_. Clearly, Ru_1_/N‐C_Pores_ exhibited a low H_2_O_2_ yield, which was less than 3.5%, and the calculated electron transfer number (n) was close to four between the potential range of 0.15–0.7 V versus RHE, indicating a desirable 4e^−^ transfer pathway (Figure 4i). The long‐term stability of catalysts was evaluated by accelerated degradation tests (ADTs) in low (0.6–1.0 V vs RHE) and high (1.0–1.5 V vs RHE) potential ranges for 20 000 cycles. After low potential ADTs, Ru_1_/N‐C_Pores_ showed only 18 mV loss in *E*_1/2_, superior to that of commercial Pt/C (24 mV, Figure S13, Supporting Information). Following high potential ADTs, a relatively severe decay was occurred, with commercial Pt/C suffering a 40 mV decline in *E*_1/2_, whereas Ru_1_/N‐C_Pores_ suffered merely a 29 mV *E*_1/2_ loss (Figure S14, Supporting Information). As illustrated in Figure S15 (Supporting Information) and detailed in Tables S7–S9 (Supporting Information), the *j*_k,mass_ of Ru_1_/N‐C_Pores_ experienced respective declines of 16.1% and 45.2% after low and high potential ADTs, outperforming commercial Pt/C (42.9% and 64.3%). The TEM and HAADF‐STEM images in Figure S16a–c (Supporting Information) revealed that Ru_1_/N‐C_Pores_ preserved its initial porous morphology after low potential ADTs, with isolated Ru atoms anchoring on the N‐C_Pores_, possibly owing to the strong interaction effect of doped‐N to Ru atoms which suppress Ru aggregation and dissolution. Nevertheless, after high potential ADTs, the TEM and HAADF‐STEM images of Ru_1_/N‐C_Pores_ (Figure S16d–f, Supporting Information) evidenced the disruption of the porous N‐C_Pores_ substrates, accompanied by the emergence of slight particulate aggregations. The reduction of Ru content also leads to a substantial loss of RuN_4_ active sites, precipitating a significant decline in ORR performance (Table S1, Supporting Information).

Encouraged by the enhanced ORR performance, the as‐prepared Ru_1_/N‐C_Pores_ was further evaluated in a real fuel cell device as the cathode catalyst (Figure S17, Supporting Information). With a view toward practical applications, the polarization performance was tested in a 1.5 bar humidified H_2_/air system (80 °C). The peak power density of 0.40 W cm^−2^ was achieved for the cell with Ru_1_/N‐C_Pores_ cathode, representing 66% of the value based on commercial Pt/C (0.61 W cm^−2^), which surpassed the majority of the reported Pt‐free catalysts. Finally, a durability test was performed according to DOE catalyst evaluation protocols for 30 000 ADTs cycles. The Ru_1_/N‐C_Pores_ suffered a drop of 118 mV at current density of 0.8 A cm^−2^, a performance that outstrips commercial Pt/C (134 mV); nonetheless, it still lags behind the DOE target (<30 mV) (Figure S18, Supporting Information).

### 2.3 ORR Catalytic Mechanism

**Figure 5:**
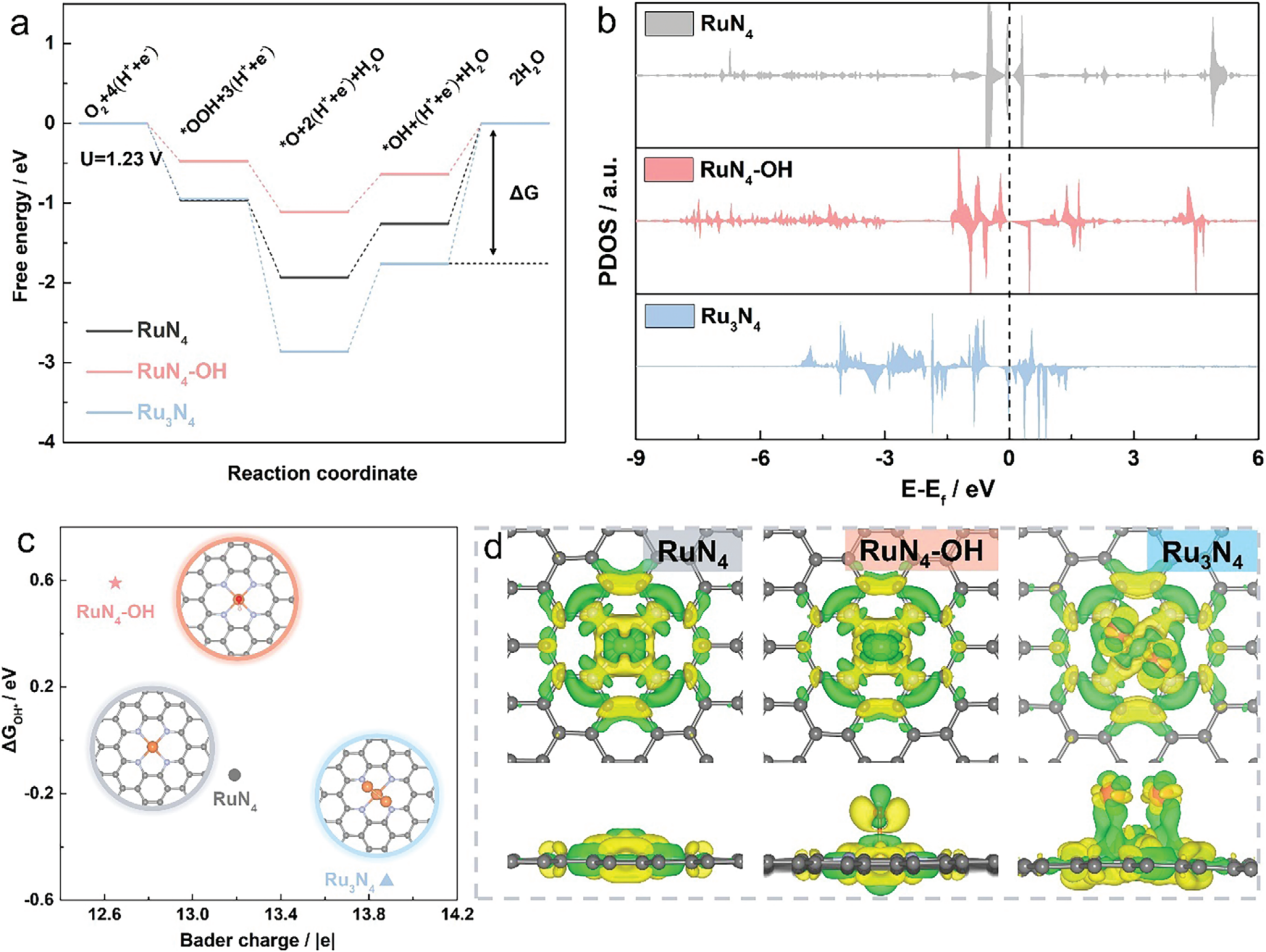
a) The calculated free energy plots of the ORR steps on RuN_4_, RuN_4_‐OH, and Ru_3_N_4_ systems at the equilibrium potential of U = 1.23 V versus RHE; b) The PDOS of Ru‐*d* in RuN_4_, RuN_4_‐OH, and Ru_3_N_4_; c) Relationship between Bader charge and *∆G*_OH*_ of single‐atom Ru in RuN_4_, RuN_4_‐OH, and Ru_3_N_4_; d) Differential charge density analysis of RuN_4_, RuN_4_‐OH and Ru_3_N_4_ (yellow and green represent electron accumulation and depletion).

DFT calculations were carried out to reveal the influence of interaction mechanism between active sites and ORR intermediates on the catalytic performance (see Supporting Information for computational methods). To assess the ORR activity, the adsorption free energy of OH^*^, denoted as *ΔG*_OH*_, was evaluated on RuN_4_, PtN_4_, and PdN_4_ systems, respectively. As shown in Figure S19 (Supporting Information), the *ΔG*_OH*_ on RuN_4_ was −0.13 eV, more negative than that on PtN_4_ (2.38 eV) and PdN_4_ (2.37 eV), which indicates that OH^*^ would spontaneously adsorb onto the RuN_4_ active sites via water dissociation.^[^ ^56^, ^57^, ^58^ ^]^ OH^*^, acting as a modifying ligand, composes the active center of the RuN_4_‐OH, a configuration that has been extensively reported for participating in various catalytic oxidation reactions.^[^ ^29^, ^48^ ^]^ To clarify the effect of adsorbed OH^*^ on boosting ORR catalytic activity, the calculated Gibbs free energies for ORR intermediates on RuN_4_, RuN_4_‐OH, PtN_4_, and PdN_4_ are listed in Table S10 (Supporting Information). Figure S20 (Supporting Information) shows the optimized adsorption configurations of OOH^*^, O^*^, and OH^*^ on RuN_4_‐OH as an example. Combined with **Figures** **5**a and S21 (Supporting Information), it reveals that at the equilibrium electrode potential (U) of 1.23 V versus RHE, the desorption of the extremely strong adsorbed OH^*^ prohibited the oxygen reduction process of RuN_4_ and RuN_4_‐OH, while the reduction step of O_2_ to form OOH^*^ was the rate‐determining step for PtN_4_ and PdN_4_. The lowest reaction barrier was achieved on RuN_4_‐OH (0.64 eV), far superior to that of RuN_4_ (1.26 eV), PtN_4_ (1.36 eV), and PdN_4_ (1.32 eV), which suggests that RuN_4_‐OH system exhibited the fastest ORR kinetics. According to the coordination configuration of Ru active sites in Ru_x_/N─C, the Ru_3_N_4_ structure was established to simulate small clusters of Ru, and its optimized adsorption configurations are shown in Figure S22 (Supporting Information). The Gibbs free energies of intermediates on Ru_3_N_4_ moiety (Figure 5a; Table S10, Supporting Information) are more negative than that of RuN_4_‐OH, suggesting that the adsorption of ORR intermediates is so strong that the reaction barrier will be significantly increased as 1.76 eV. Given the strong correlation between the d‐band center (average energy of d‐band) of metal and its interaction with adsorbates, exploring the electronic structure of the Ru active center, including the density of states (DOS), offers an insightful explanation for the outstanding ORR activity of the RuN_4_‐OH system. The lower d‐band center of RuN_4_‐OH (−1.24 eV) in comparison with RuN_4_ (−0.32 eV) and Ru_3_N_4_ (−1.22 eV) may be attributed to electron redistribution triggered by the adsorption of OH^*^ (Figure 5b). A lower d‐band center leads to the downshift of antibonding orbitals, consequently resulting in more orbital occupation, which in turn weakens the intermediate absorption energy.^[^ ^59^, ^60^ ^]^ Bader charge analysis, as depicted in Figure 5c, further elucidates that the electron deprivation facilitated by the adsorbed OH^*^ serves to enhance the high oxidation state of Ru active center, resulting in a weaker binding strength between RuN_4_‐OH and OH^*^. In addition, the charge density difference analysis of RuN_4_ and RuN_4_‐OH clearly illustrated extra charge transfer taking place at the Ru‐OH interface (Figure 5d). Thus, RuN_4_‐OH system, with spontaneous adsorbed OH^*^ as modifying ligand, is more favorable to achieve suitable intermediate (OOH^*^, O^*^, OH^*^) binding energy by modulating the electronic structure of the active center.

## 3 Conclusion

In conclusion, the molten salt‐assisted synthesis strategy was used in the fabrication of highly porous PM‐SACs (PM = Ru, Pt, and Pd), including the formation of hierarchical porous N‐doped carbon substrate (N‐C_Pores_) and the subsequent loading of isolated PM atoms (PM_1_/N‐C_Pores_). RRDE and PEMFCs tests reveal that the Ru_1_/N‐C_Pores_ exhibit appreciable activity and stability, which can be attributed to its hierarchical porous structure and abundant catalytically accessible Ru‐N_4_ sites with high intrinsic activity. Structural characterizations and theoretical calculations demonstrated that the RuN_4_ configuration induces the spontaneous adsorption of OH^*^ modifying ligand, thereby regulating the electronic structure of the Ru active center to obtain the optimal binding energy of ORR intermediates. These findings provide guidance for realizing the practical application of PM‐SACs as ORR electrocatalyst in acidic media.

## Conflict of Interest

The authors declare no conflict of interest.

## Supporting information



Supporting Information

## Data Availability

The data that support the findings of this study are available from the corresponding author upon reasonable request.
