# Supplementary material for: Molten Salt‐Assisted Synthesis of Porous Precious Metal‐Based Single‐Atom Catalysts for Oxygen Reduction Reaction
Source: Adv Sci (Weinh). 2024 Dec 31;12(8):2410784. doi: 10.1002/advs.202410784 (PMC11848534; doi:10.1002/advs.202410784)
Supplement: Supplementary file 1 — Supporting Information [file ADVS-12-2410784-s001.docx]

Supporting Information

**Molten Salt-Assisted Synthesis of Porous Precious Metal-Based Single-Atom Catalysts for Oxygen Reduction Reaction**

Chenming Fan^a,b^, Xin Gao^a^, Pengyi Tang^b,c*^, Qiang Wang^b*^, and Bing Li^,a*^

^a^ School of Mechanical and Power Engineering, East China University of Science and Technology, Shanghai 200237, People’s Republic of China

^b^ 2020 X-Lab, Shanghai Institute of Microsystem and Information Technology, Chinese Academy of Sciences, Shanghai 200050, People’s Republic of China

^c^ National Key Laboratory of Materials for Integrated Circuits, Shanghai Institute of Microsystem and Information Technology, Chinese Academy of Sciences, Shanghai, 200050, People’s Republic of China

*Corresponding authors:

bingli@ecust.edu.cn (Bing Li); wangqiang@mail.sim.ac.cn (Qiang Wang); py.tang@mail.sim.ac.cn (Pengyi Tang)

**Experimental Section**

*Chemicals and reagents*: Zn(NO_3_)_3_·6H_2_O, Ru(acac)_3_, Pt(acac)_2_, Pd(acac)_2_ and 2-methylimidazole were used as received from Sigma-Aldrich and RuCl_3_, PtCl_4_, PdCl_2_ and NaCl were purchased from Aladdin. All other chemicals and solvents were used directly without further purification.

*Synthesis of ZIF-8 and PM@ZIF-8*: Zn(NO_3_)_3_·6H_2_O (12.5 mmol) and PM(acac)_n_ (PM=Ru, Pt, Pd, 0.4 mmol) were mixed in 100 mL of methanol. After sonication, the solution was then added into a solution of methanol containing 2-methylimidazole (100 mmol). The PM@ZIF-8 was achieved by centrifugation after stirred vigorously for 6 h and aged for 6h. The ZIF-8 was prepared under the same conditon without adding PM(acac)_n_. All products were washed with methanol, then dried at 80℃ under vacuum.

*Synthesis of N-C and PM_x_/N-C*: The ZIF-8 and PM@ZIF-8 precursors were transferred into a quartz boat, and then heated at 950 ºC under a 5% H_2_/Ar flow for 1 h. The product was washed with HCl (1 M) at 60 ºC to remove unstable species.

*Synthesis of N-C_Pores_*: ZIF-8 powder and NaCl with a mass ratio of 1:1, 1:2 and 1:4 were mixed by ball milling for 1 h, after which the mixture were heated at 950 ºC under a 5% H_2_/Ar flow for 1 h. A series of N-C_Pores_ supports, designated as N-C_Pores_(1:1), N-C_Pores_ and N-C_Pores_(1:4), were achieved after washed with deionized water to remove any residual NaCl, then dried at 80℃ under vacuum.

*Synthesis of PM_1_/N-C_Pores_:* 100mg of N-C_Pores_(1:1), N-C_Pores_ and N-C_Pores_(1:4) were dispersed in 40 mL methanol solution, respectively. After ultrasound for 30 min, a uniform slurry was obtained, and then 10 mL of PMCl_n_ methanol solution was added to it drop by drop. The mixed solution was continuously stirred at room temperature for 12 h, and the precipitate obtained by centrifugation was washed with methanol and dried under vacuum at 80℃. The as-obtained powder was heated at 950 ºC under a 5% H_2_/Ar flow for 1 h, and then washed with HCl (1 M) at 60 ºC to remove unstable species. The products were donated as PM_1_/N-C_Pores_(1:1)、PM_1_/N-C_Pores_、PM_1_/N-C_Pores_ (1:4), respectively.

*Characterizations*: Powder X-ray diffraction (XRD) was performed with a rotating anode X-ray powder diffractometer, using a Cu anode radiation source. Scanning electron microscopy (SEM) measurements were carried out on a Hitachi S-4800 field emission scanning electron microscope. The elemental analysis of products was performed through inductively coupled plasma emission spectroscopy (ICP-OES) on Agilent 5110. The ICP-OES samples were prepared by leaching the samples a concentrated HCl/HNO_3_ (3/1) solution at 70℃ and then diluting it with water. 5 standard samples were measured at the same time to prepare calibration curves. Transmission electron microscopy (TEM) images were obtained from a Talos F200X G2 TEM operating at an acceleration voltage of 200keV. Aberration-corrected scanning transmission electron microscopy (HAADF-STEM) images were achieved on a Themis Z TEM/STEM operating at an acceleration voltage of 300keV. Raman spectra were collected on Thermo Scientific DXR with laser wavelength of 532 nm at an incident power density of 5 mW cm^-2^ to investigate the change in the molecular polarization and associated scattering frequencies of the samples. Brunauer-Emmett-Teller (BET) surface area was determined using N_2_ adsorption/desorption isotherms achieved on Micromeritics ASAP 2460, and the pore size distribution was computed using the non-local density functional theory method. X-ray photoelectron spectroscopy (XPS) measurements was performed on a Thermo Scientific Theta Probe instrument with an Al Kα (1486.7 eV) radiation source. The charge corrections and binding energies of all elements were compared with the C1s binding energy of exogenous carbon (BE≈284.8 eV). X-ray absorption spectroscopy (XAS) was performed at BL14W1 station in Shanghai Synchrotron Radiation Facility (SSRF). Ru foil and RuO_2_ samples were used as references.

*Electrochemical measurements*: In this paper, all electrochemical measurements were performed in a three-electrode cell with CHI 760e electrochemical workstation. A glassy carbon rotating ring-disk electrode (RRDE, 5.6 mm in diameter) modified with the catalyst, served as the working electrode; a Hg/Hg_2_SO_4_ electrode calibrated against the reversible hydrogen electrode (RHE) fulfilled the role of the reference electrode; and a Pt wire (0.5 mm in diameter) was employed as the counter electrode. 10 mg as-prepared catalysts were uniformly dispersed in 990 μL of isopropyl alcohol and 10 μL of 5 wt.% Nafion solution after ultrasonicated for 30 min. The catalyst thin film coated RRDE electrode with a loading of 0.3 mg cm^-2^ was formed by drying in air. For comparison, a commercial 20 wt.% JM Pt/C coated RRDE electrode with the mass loading of 15 μg_Pt_ cm^-2^ was selected.

All ORR tests were performed in 0.1 M HClO_4_ electrolyte. Cyclic Voltammetry curves (CVs) were recorded in the potential range of 0.95 to 1.05 V vs RHE, and the double-layer capacitance (*C*_dl_) was determined based on the linear fitting slope of the current to scan rate. The calculated *C*_dl_ were converted into electrochemical surface area (ECSA) by the following equation:

$\text{ECSA=}\frac{\text{C}_{\text{dl}}}{\text{C}_{\text{s}}}$ (1)

where *C*_s_ is the specific capacitance (the average specific capacitance for a flat electrode is 0.04 mF cm^-2^). ORR polarization curves between 0.15 and 1.1 V vs. RHE were recorded at 10 mV s^-1^ in O_2_-saturated 0.1 M HClO_4_ electrolyte with a rotation speed of 1600 rpm. The ORR kinetic current density (*j*_k_) excluding the mass-transfer effect was evaluated based on Koutecky-Levich equation:

1/*j*=1/*j*_k_+1/*j*_l_ (2)

where *j* and *j*_l_ represent the measured and diffusion-limiting current density, respectively. The turnover frequency (TOF) is estimated by:

$\text{TOF}\text{=}\frac{\text{j}_{\text{k}}\text{×}\text{ }\text{N}_{\text{e}}}{\text{m}_{\text{Ru}}\text{×}\text{N}_{\text{A}}/\text{M}_{\text{Ru}}}$ (3)

where *N*_e_ is electron number per Coulomb, *m*_Ru_ is the mass loading of metal on the electrode, *N*_A_ is Avogadro constant and *M*_Ru_ is molar mass of Ru. The mass activity (*j*_k,mass_) of the catalysts were then calculated by combining the *j*_k_ and metal loading. The number of electrons transferred (*n*) and H_2_O_2_ selectivity (HO_2_^-^%) were calculated by the following equations:

$\text{n=}\frac{\text{4N}\text{I}_{\text{d}}}{\text{N}\text{I}_{\text{d}}\text{+}\text{I}_{\text{r}}}$ (4)

$\text{HO}_{\text{2}}^{\text{-}}\text{\%=}\frac{\text{200I}_{\text{r}}}{\text{N}\text{I}_{\text{d}}\text{+}\text{I}_{\text{r}}}$ (5)

where *I*_d_ is the disk current and *I*_r_ is the ring current. The current collection efficiency (N) of RRDE is calibrated to be 0.36. Accelerated durability tests (ADTs) were further applied to evaluate the stability of the as-prepared catalysts, by cycling in low (0.6-1.0 V vs. RHE, 100 mV s^-1^) and high (1.0-1.5 V vs. RHE, 500 mV s^-1^) potential ranges.

*Membrane electrode assemblies (MEAs) fabrication and fuel cell tests:* The catalyst was mixed with Nafion ionomer solution diluted by water/isopropanol and sprayed onto a 5-cm^2^ Nafion NC700 membrane after ultrasonicating. All anode catalyst layers were endowed with a Pt loading of 0.12 mg_Pt_ cm^-2^. A catalyst loading of 4 mg_Pt_ cm^-2^ was selected for the cathode catalyst layers and compared with the Pt loading of 0.12 mg_Pt_ cm^-2^. To construct the final MEA, the cathode and anode gas diffusion layers (Avcarb, GDS3260) were hot-pressed onto a prepared membrane at 120℃ under a pressure of 5.5 MPa. Fuel cell evaluations were carried out in a single cell utilizing a Scribner 850e fuel cell test station. All cells were operated in a humidified H_2_/air (100%RH, 600/1500 sccm) system at 80℃ with a back pressure of 1.5 bar. Accelerated durability tests (ADTs) were operated between 0.6-1.0 V, using triangle wave cycling (100 mV s^-1^).

*Density functional theory (DFT) computational methods*: DFT calculations were performed by using the Vienna Ab initio simulation package (VASP).^[1, 2]^ We employed the Perdew-Burke-Ernzerh (PBE) functional within the framework of the generalized gradient approximation (GGA) to described the exchange-correlation potential.^[3]^ The ionic cores were modeled using the projector augmented wave (PAW) method, while the valence electrons were described with a plane wave basis set, applying a kinetic energy cutoff of 500 eV.^[4]^ Convergence in structural optimization was achieved when the force on each atom was less than 0.02 eV Å^-1^, ensuring a stable configuration. Dispersion interactions were described by imposing Grimme’s DFT-D3.^[5]^ To construct PMN_4_ (PM=Ru, Pt, Pd) structure, two carbon atoms were replaced by one PM atom in a 5×5 supercell graphene layer, and then four nitrogen atoms superseded the four carbon atoms adjacent to PM atom. A vacuum region of 20 Å along z-direction was used to avoid the periodic interactions. Based on the Monkhorst-Pack grid method, a k-point mesh of 3×3×1 was applied for geometric optimization.^[6]^ All structure models were displayed by using VESTA.^[7]^

We consider the desirable four-electron ORR pathway as following:

$\text{O}_{\text{2}}\text{+(}\text{H}^{\text{+}}\text{+}\text{e}^{\text{-}}\text{)+*=}\text{OOH}^{\text{*}}$ (6)

$\text{OOH}^{\text{*}}\text{+(}\text{H}^{\text{+}}\text{+}\text{e}^{\text{-}}\text{)=}\text{O}^{\text{*}}\text{+}\text{H}_{\text{2}}\text{O}$ (7)

$\text{O}^{\text{*}}\text{+(}\text{H}^{\text{+}}\text{+}\text{e}^{\text{-}}\text{)=}\text{OH}^{\text{*}}$ (8)

$\text{OH}^{\text{*}}\text{+(}\text{H}^{\text{+}}\text{+}\text{e}^{\text{-}}\text{)=}\text{H}_{\text{2}}\text{O+*}$ (9)

The Gibbs free energy difference ($\text{∆}\text{G}_{\text{ad}}$) for adsorption of OOH^*^, O^*^ and OH^*^ was calculated by:^[8, 9]^

$\text{∆}\text{G}_{\text{ad}}\text{=}\text{∆}\text{E}_{\text{ad}}\text{+}\text{∆}\text{ZPE}_{\text{ad}}\text{-}\text{T∆}\text{S}_{\text{ad}}\text{+}\text{(∆}\text{G}_{\text{U}}\text{+}\text{∆}\text{G}_{\text{PH}}\text{)}$ (10)

where $\text{∆}\text{E}_{\text{ad}}$ and $\text{∆}\text{ZPE}_{\text{ad}}$ represents binding energies of adsorbates and the corrected zero-point energy, respectively. $\text{T∆}\text{S}_{\text{ad}}$ is the change in entropy at room temperature (T=298.15 K). $\text{∆}\text{G}_{\text{U}}=\text{-neU}$ is the electrons transfer effect in the electrode, where *e* is the transferred electron number and *U* is the applied potential against RHE. The pH effect ($\text{∆}\text{G}_{\text{PH}}\text{=-}\text{kBTln}\text{[}\text{H}^{\text{+}}\text{]}$) was also considered.


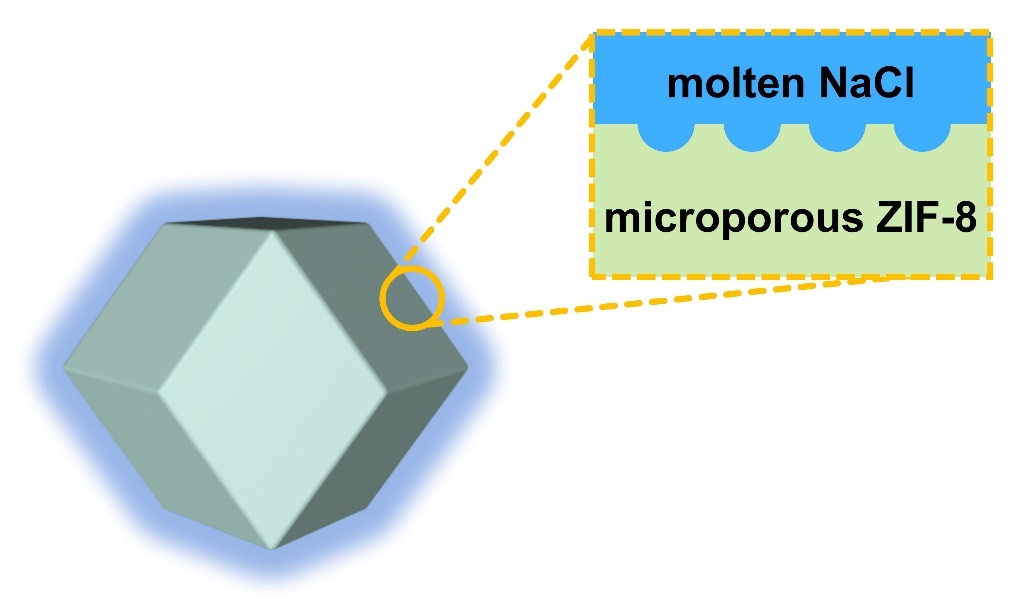


**Figure S1.** Schematic illustration of molten NaCl helping to preserve the microporous structure during pyrolysis process.


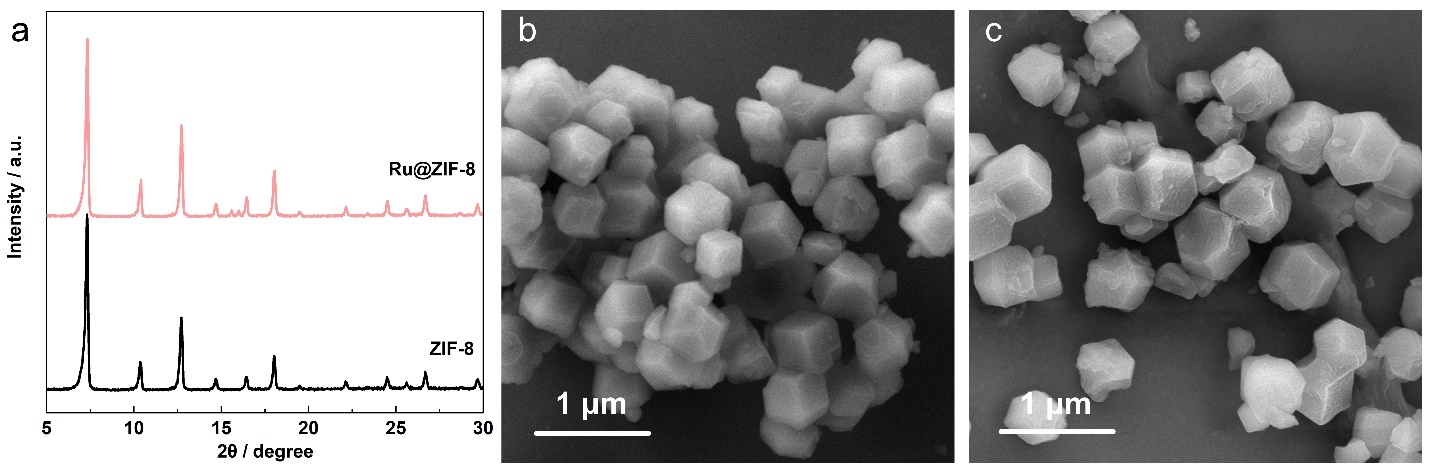


**Figure S2.** a) XRD patterns of ZIF-8 and Ru@ZIF-8; b) SEM image of ZIF-8; c) SEM image of Ru@ZIF-8.


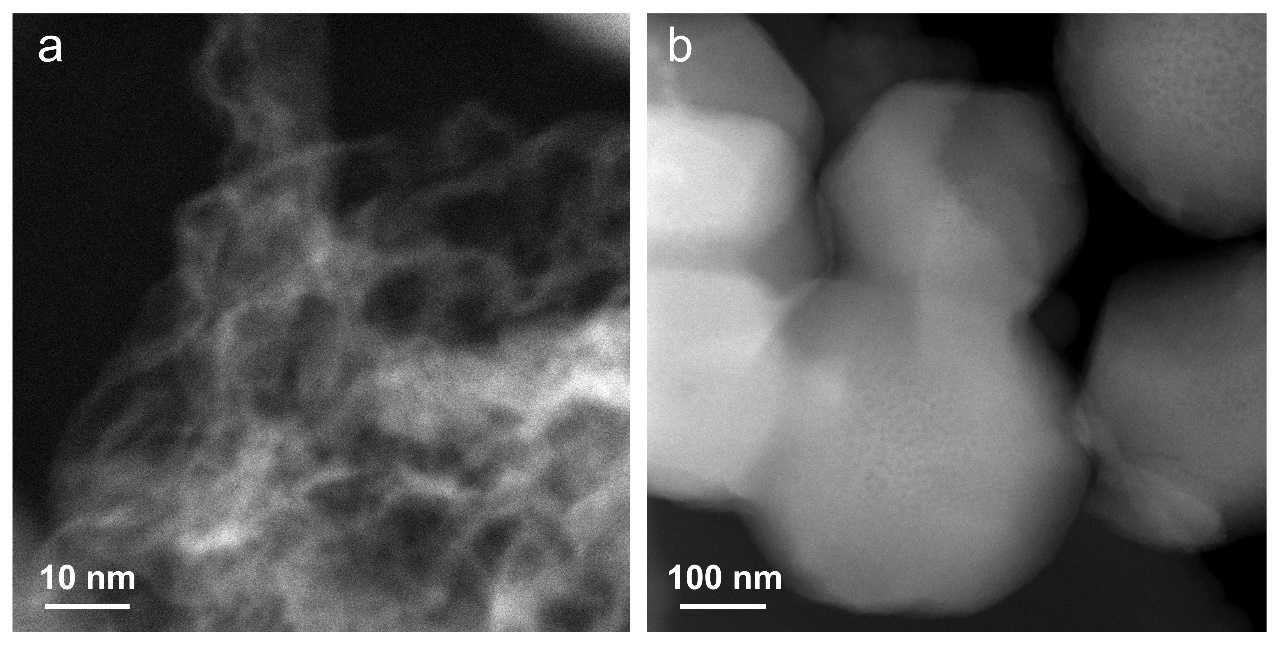


**Figure S3.** a) HAADF-STEM image of N-C_Pores_; b) HAADF-STEM image of N-C.


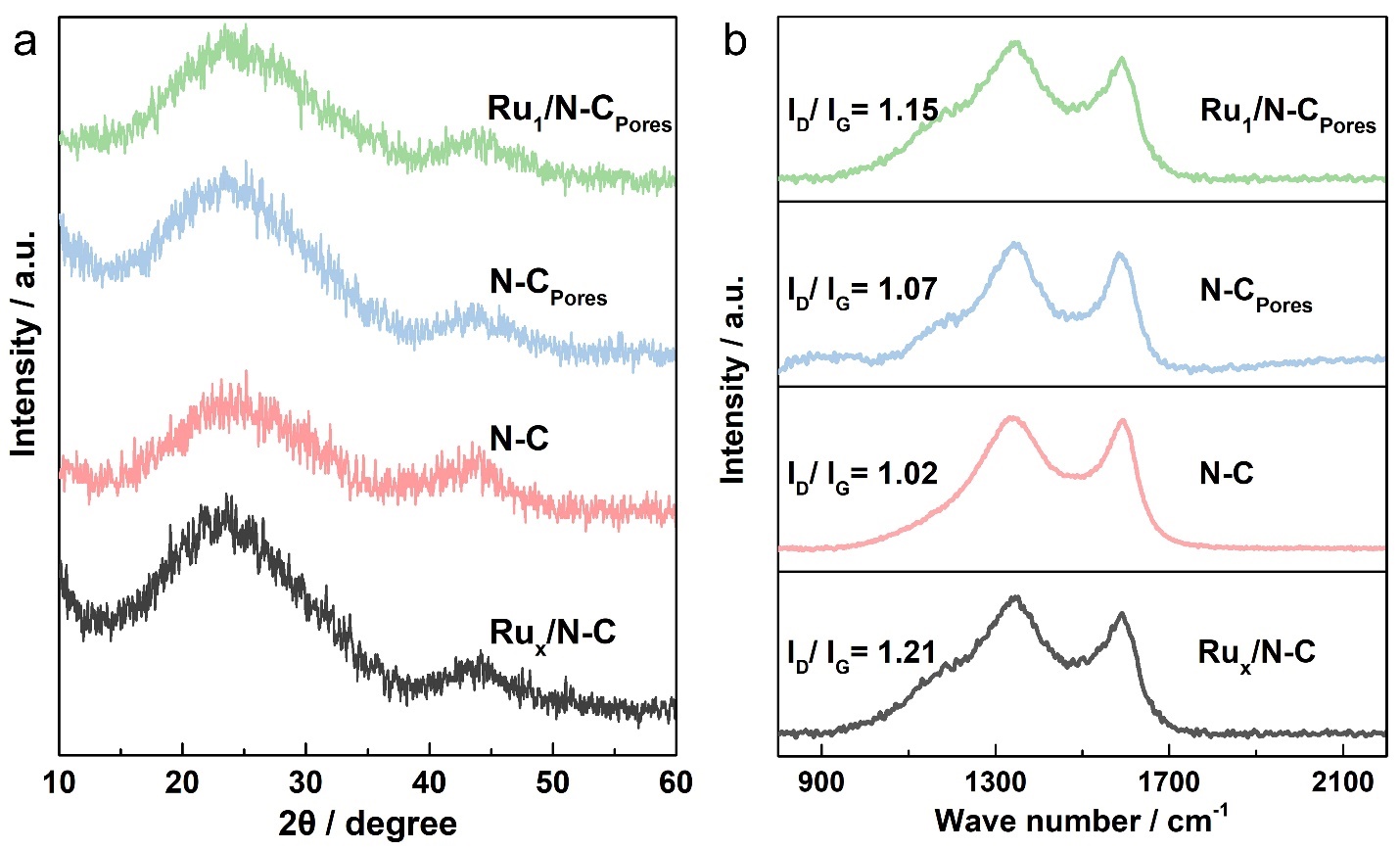


**Figure S4.** Characterization of Ru_x_/N-C, N-C, N-C_Pore_ and Ru_1_/N-C_Pores_: a) XRD patterns; b) Raman spectra.


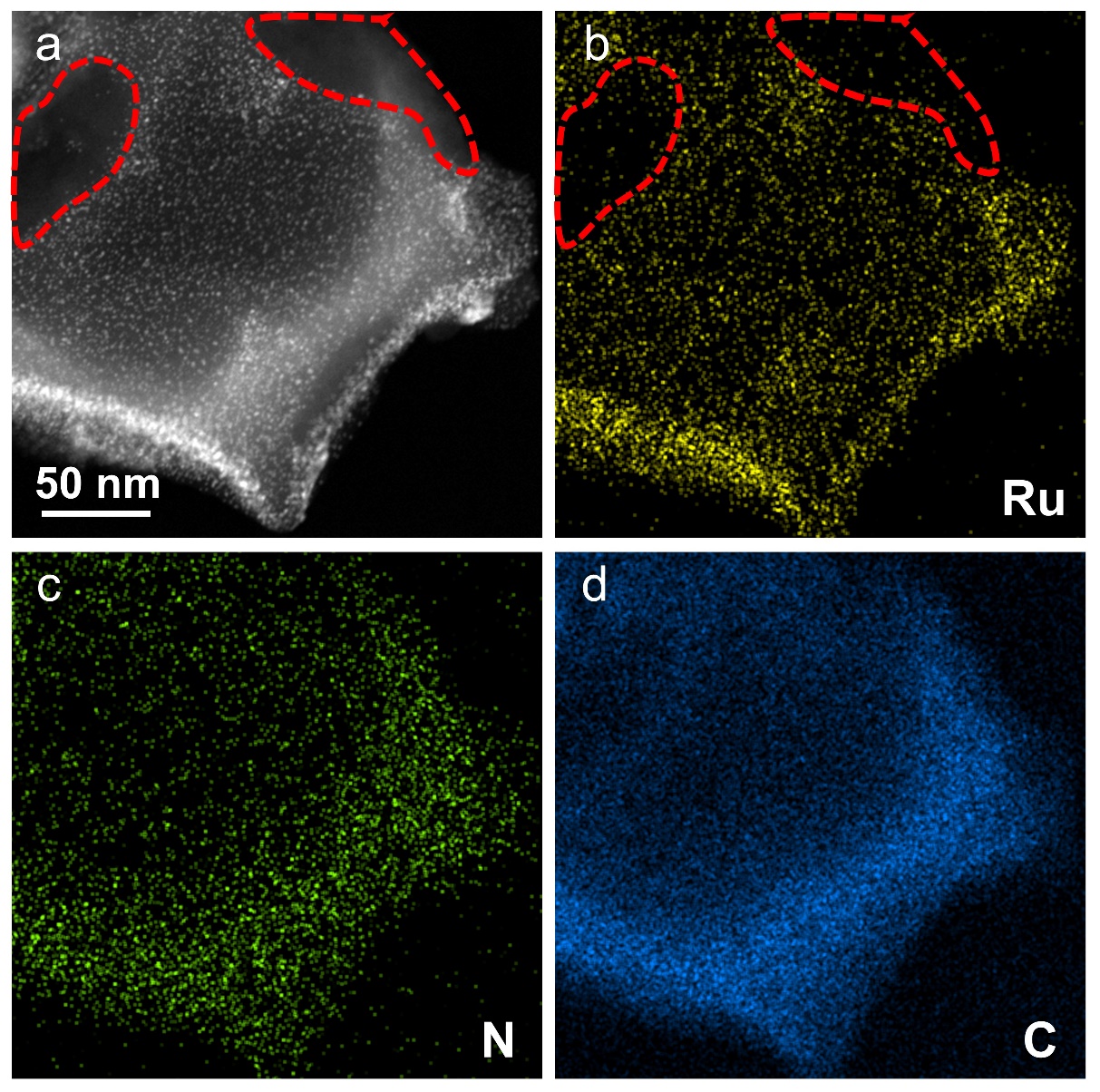


**Figure S5.** HAADF-STEM image and corresponding EDS elemental mapping of Ru_x_/N-C.

**
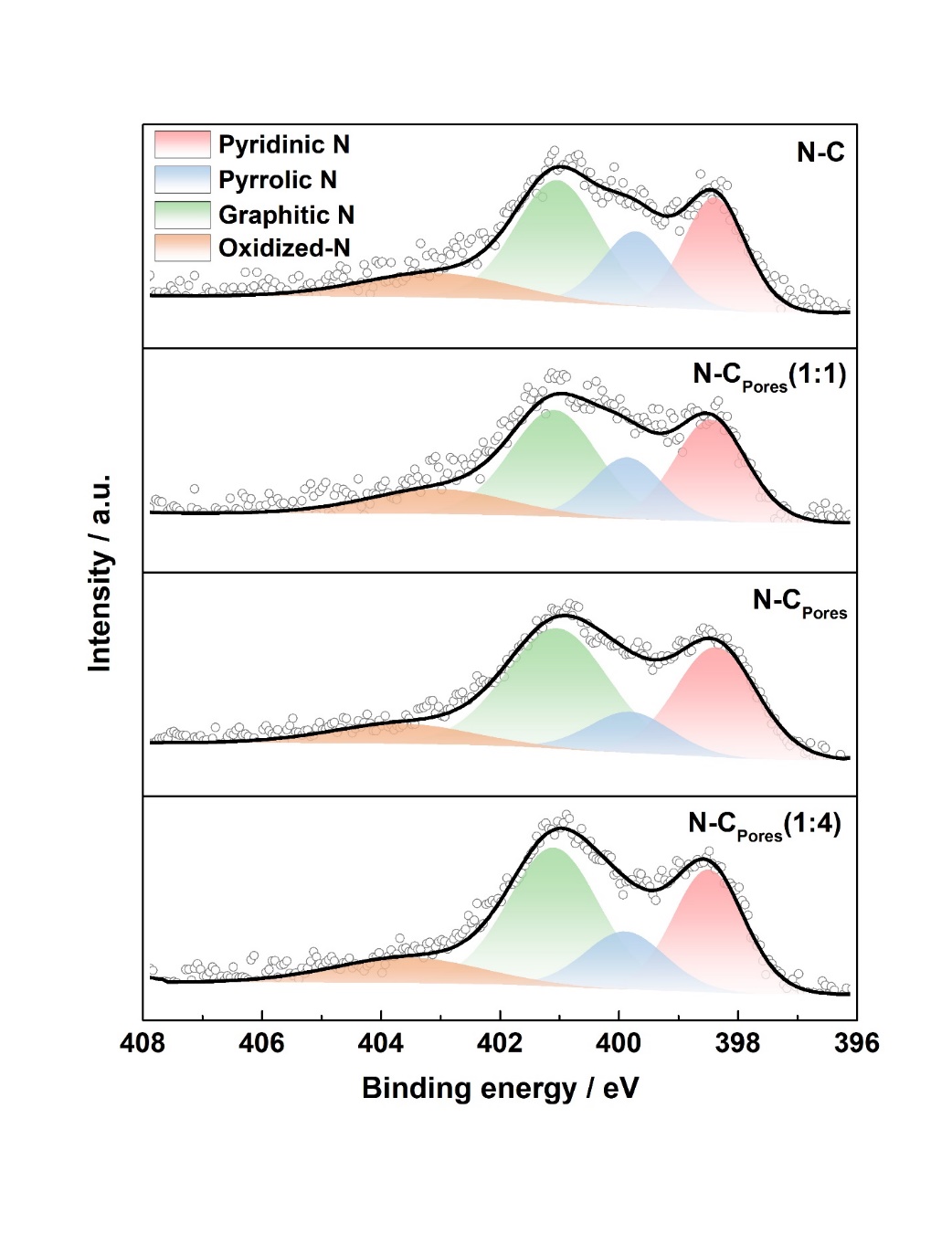
**

**Figure S6.** High-resolution N 1s XPS spectra of N-C, N-C_Pores_(1:1), N-C_Pores_ and N-C_Pores_(1:4).


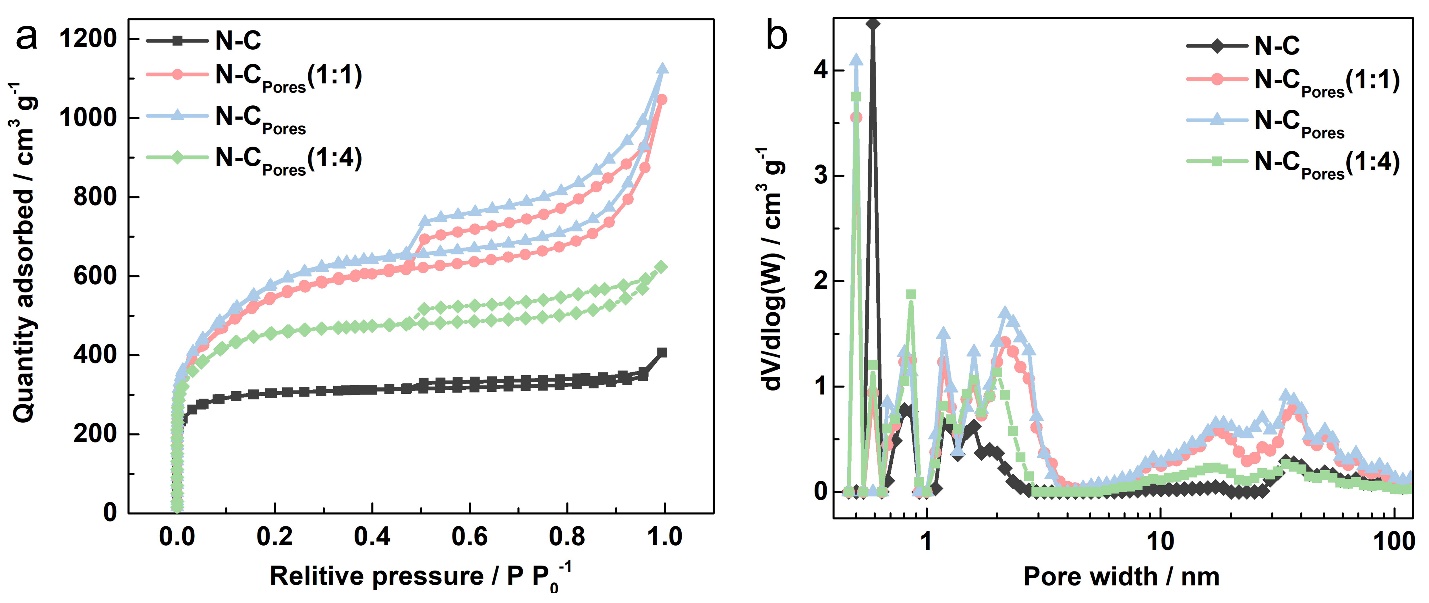


**Figure S7.** a) N_2_ adsorption/desorption isotherms and b) the corresponding pore size distribution curves of N-C、N-C_Pores_(1:1)、N-C_Pores_ and N-C_Pores_(1:4).


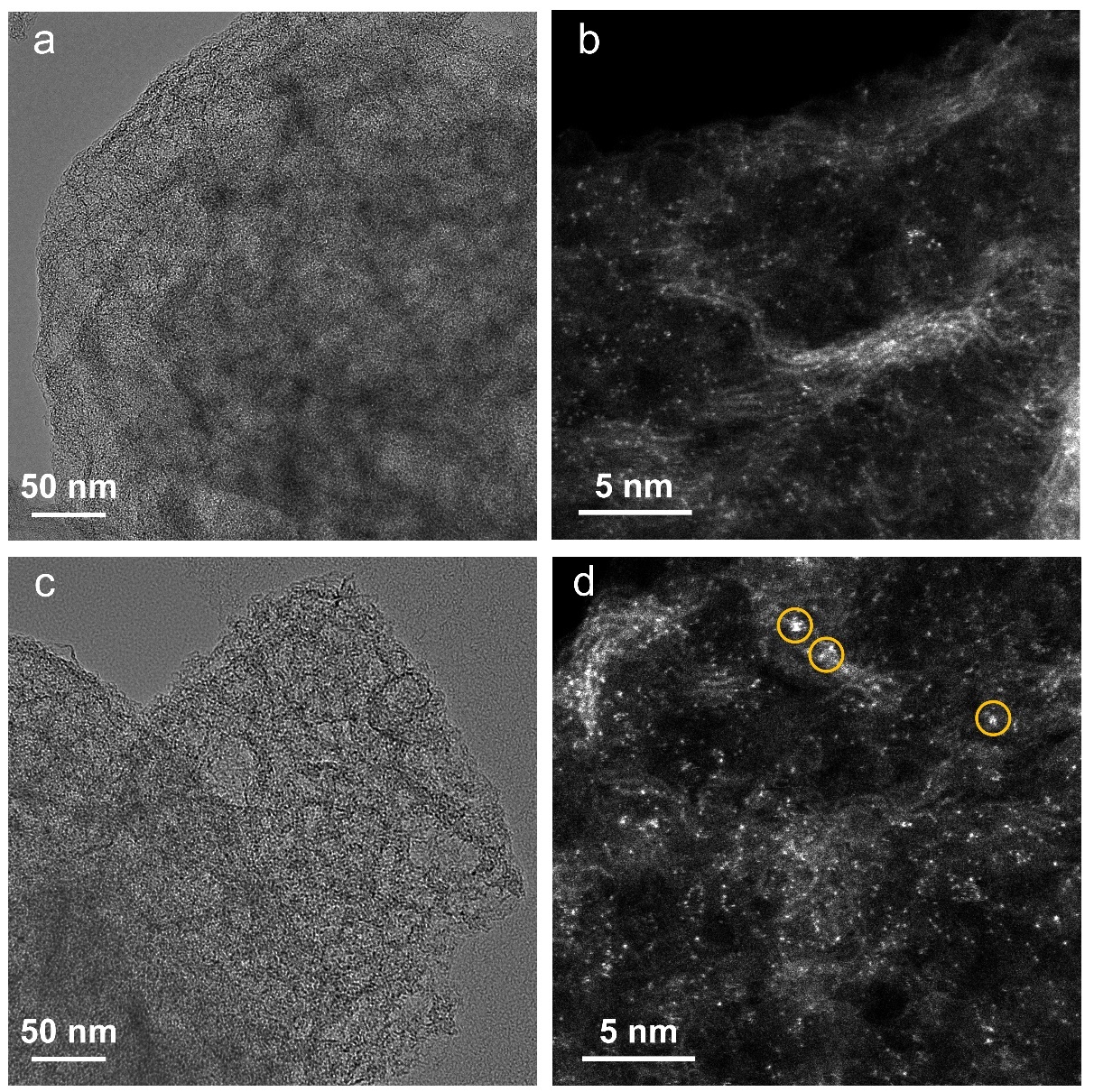


**Figure S8.** Morphology characterization of Ru_1_/N-C_Pores_(1:1): a) TEM image; b) Atomic-resolution HAADF-STEM image; Morphology characterization of Ru_1_/N-C_Pores_(1:4): c) TEM image; d) Atomic-resolution HAADF-STEM image.


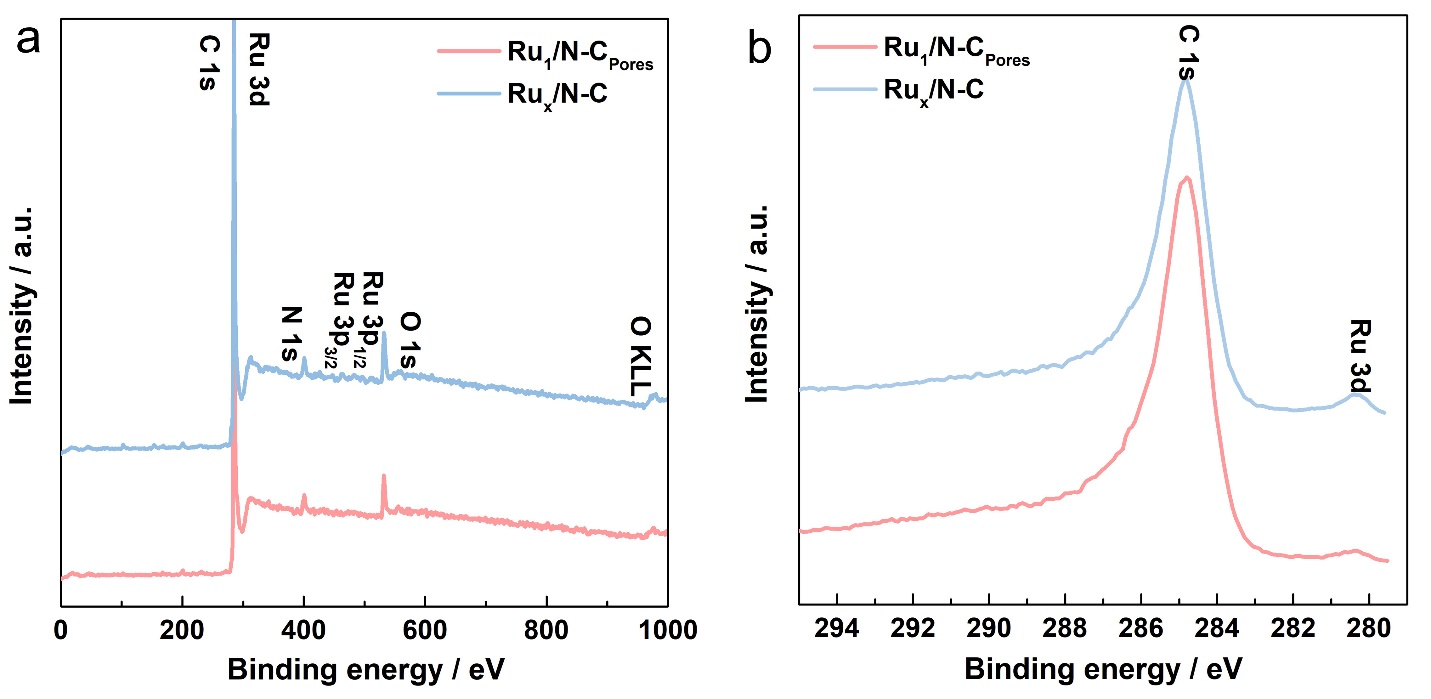


**Figure S9.** XPS spectra of Ru_1_/N-C_Pores_ and Ru_x_/N-C: a) XPS survey spectra; b) High-resolution C 1s and Ru 3d XPS spectra.

**
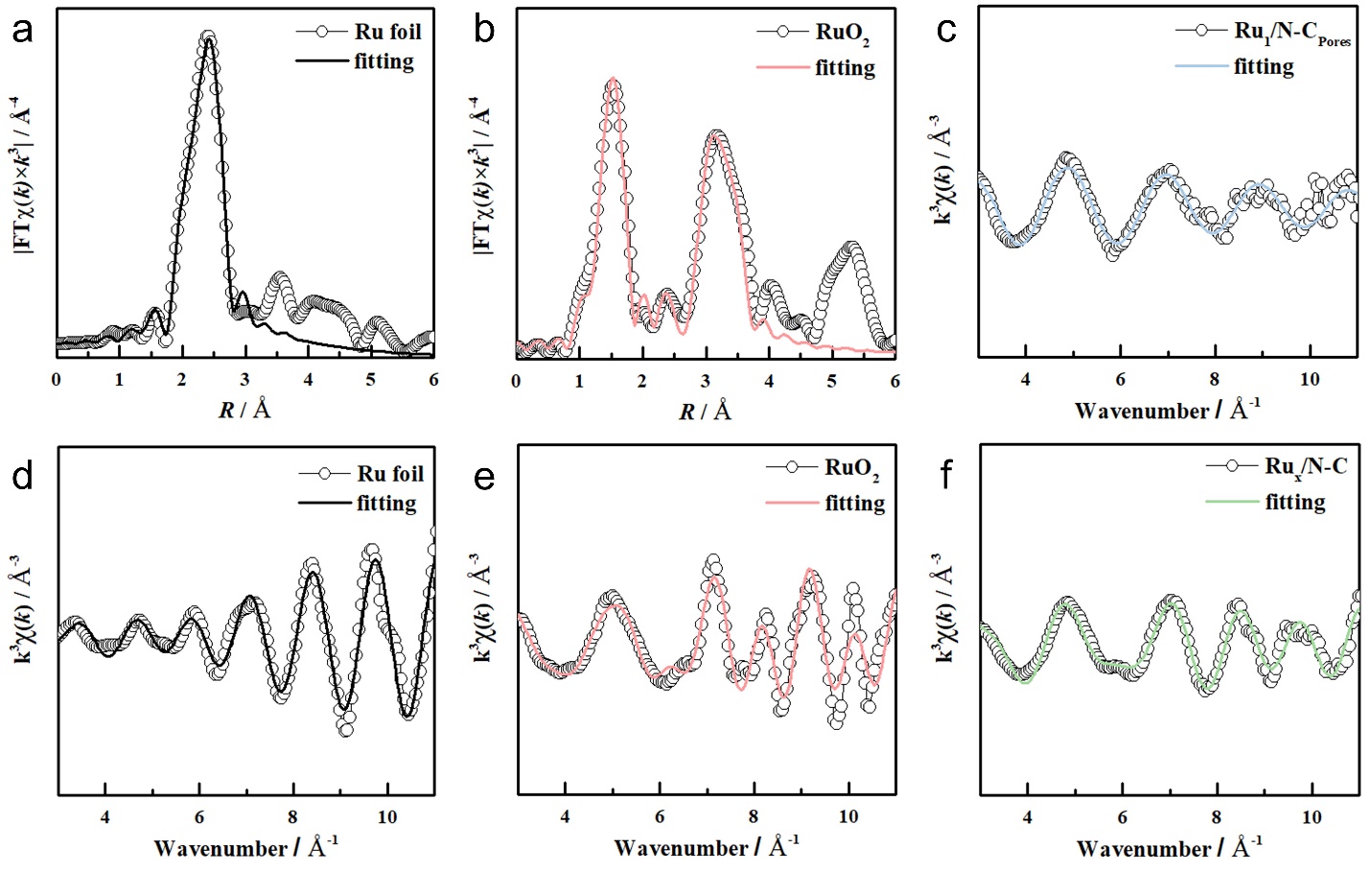
**

**Figure S10.** Ru K-edge EXASF and curve fitting for Ru foil, RuO_2_, Ru_1_/N-C_Pores_ and Ru_x_/N-C: a, b) *R*-space; c-f) *k*-space.


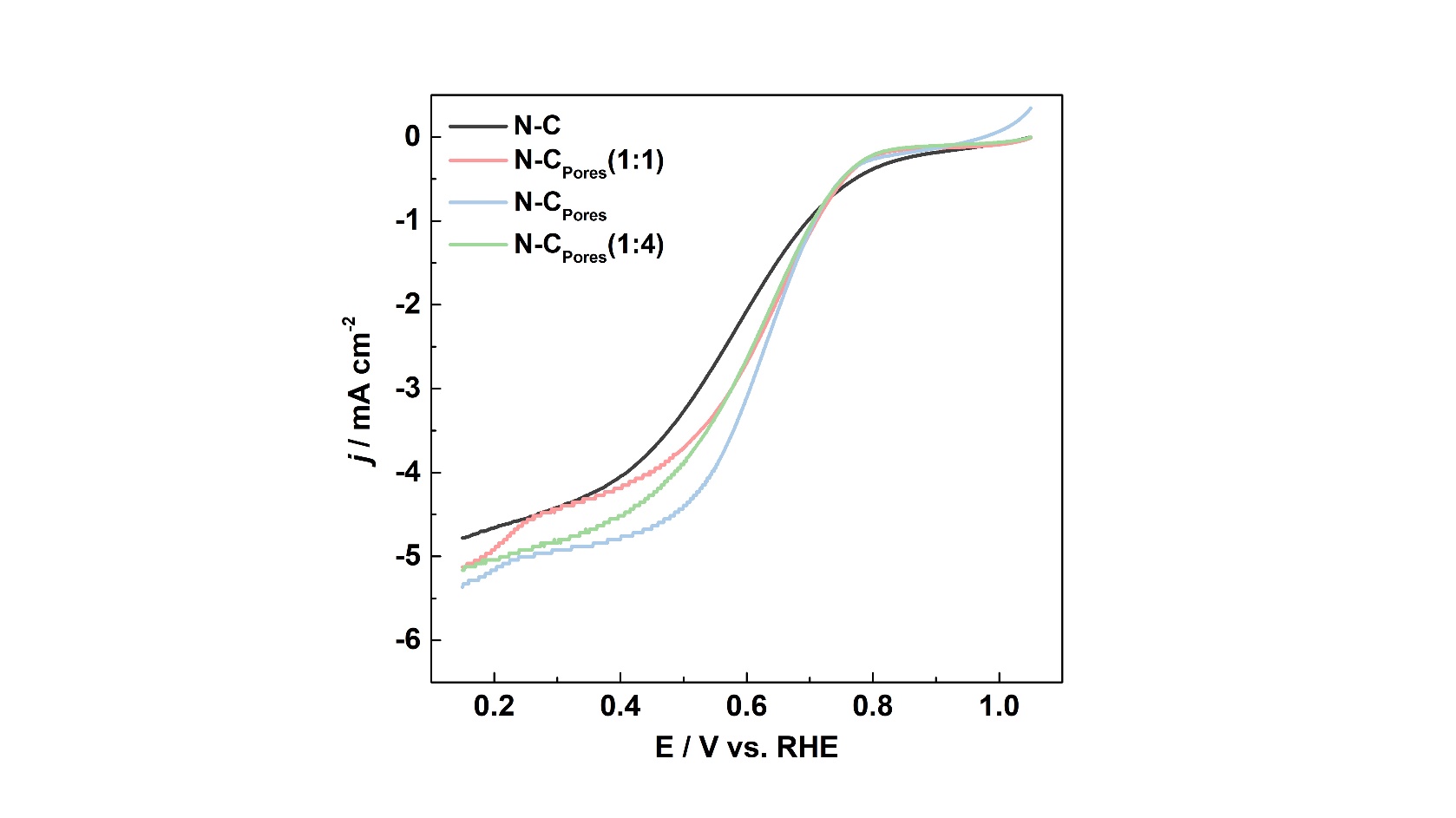


**Figure S11.** ORR polarization curves recorded in O_2_-saturated 0.1M HClO_4_ electrolyte.


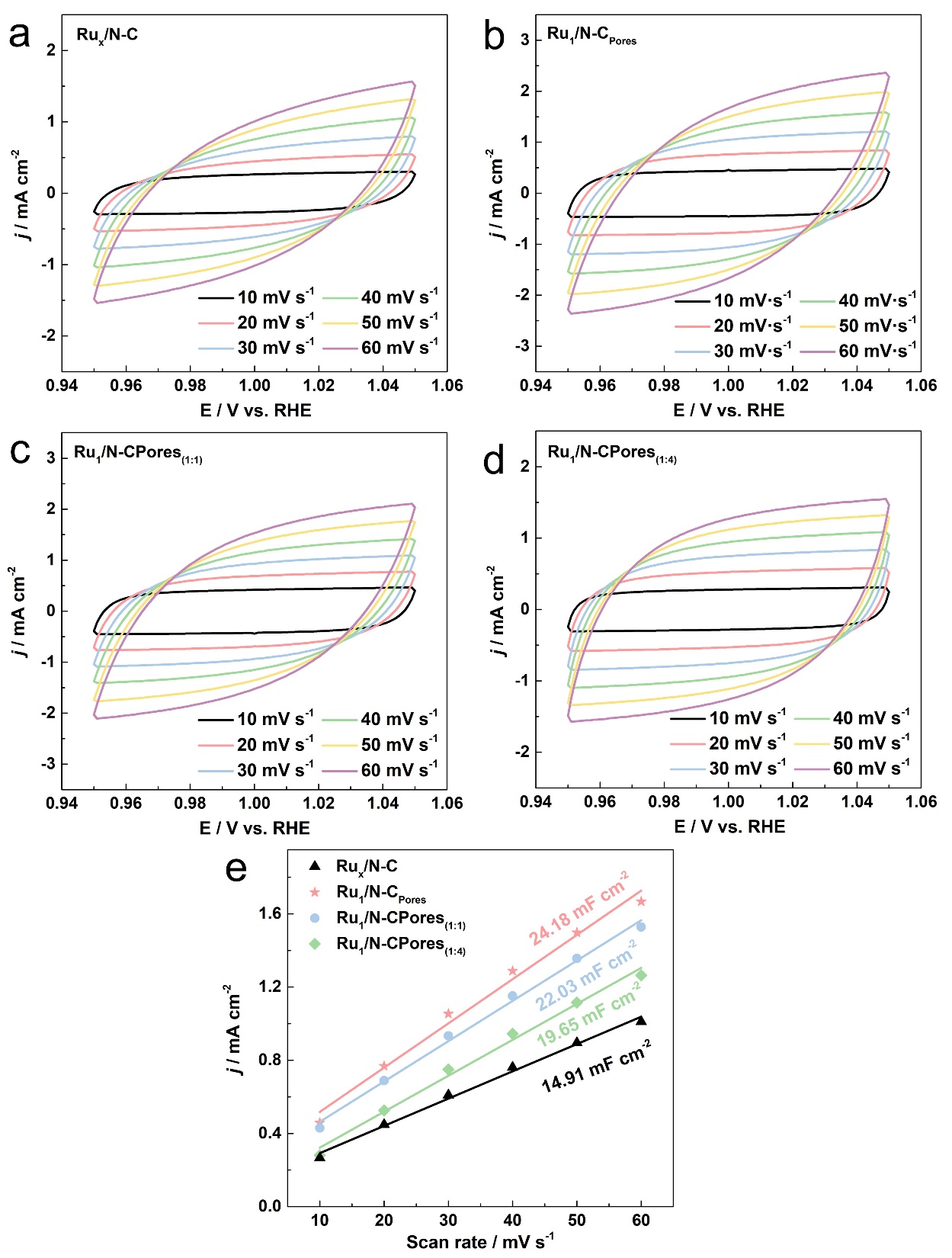


**Figure S12.** CV curves recorded at different scan rates in the non-faradaic potential range of 0.95-1.05 V vs. RHE: a) Ru_x_/N-C; b) Ru_1_/N-C_Pores_. c) Ru_1_/N-C_Pores_(1:1); d) Ru_1_/N-C_Pores_(1:4); e) C_dl_ value calculated from a-d).


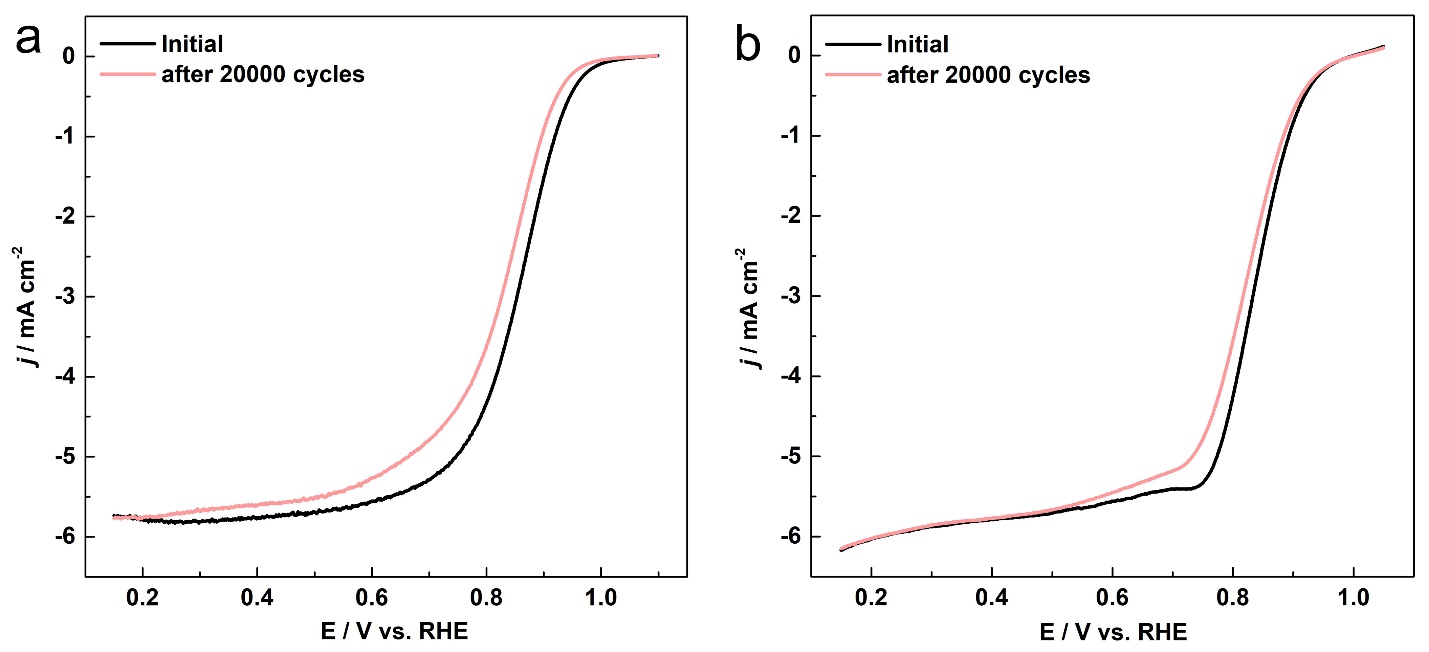


**Figure S13.** ORR polarization curves recorded in O_2_-saturated 0.1M HClO_4_ electrolyte before and after ADTs between 0.6-1.0 V vs RHE: a) Commercial Pt/C; b) Ru_1_/N-C_Pores_.


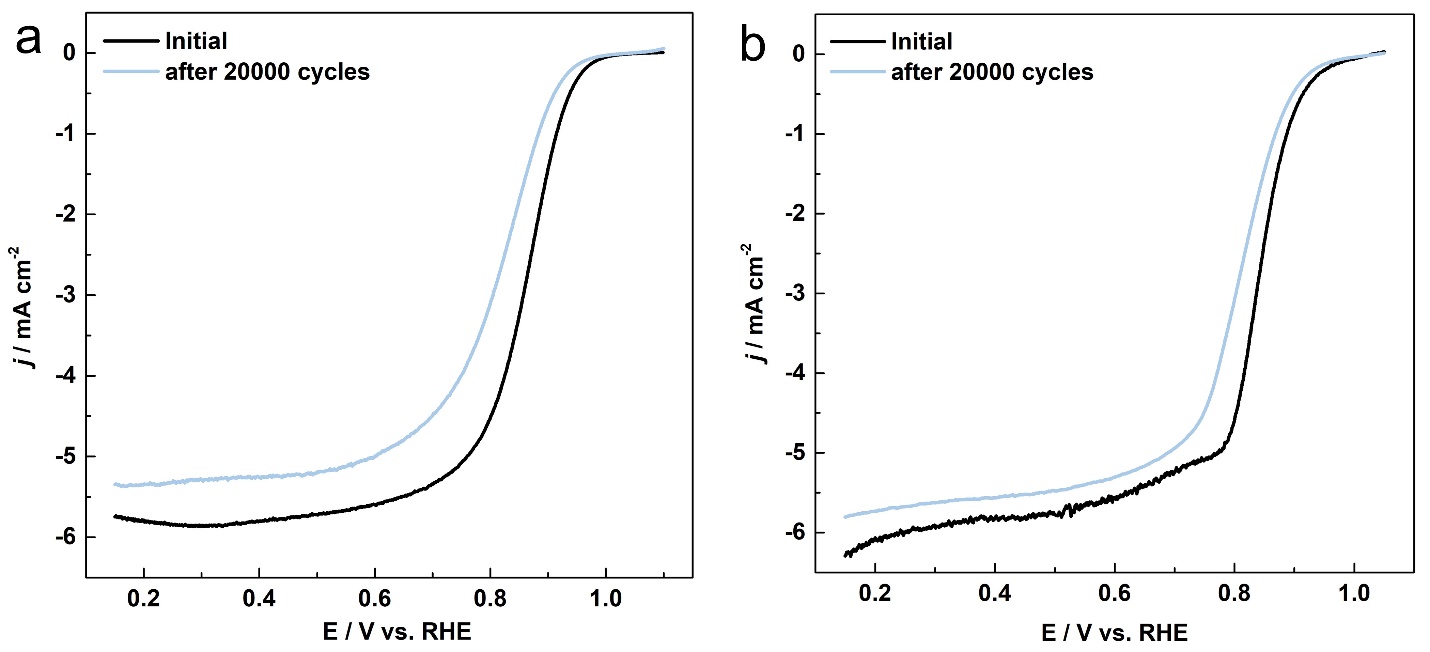


**Figure S14.** ORR polarization curves recorded in O_2_-saturated 0.1M HClO_4_ electrolyte before and after ADTs between 1.0-1.5 V vs RHE: a) Commercial Pt/C; b) Ru_1_/N-C_Pores_.


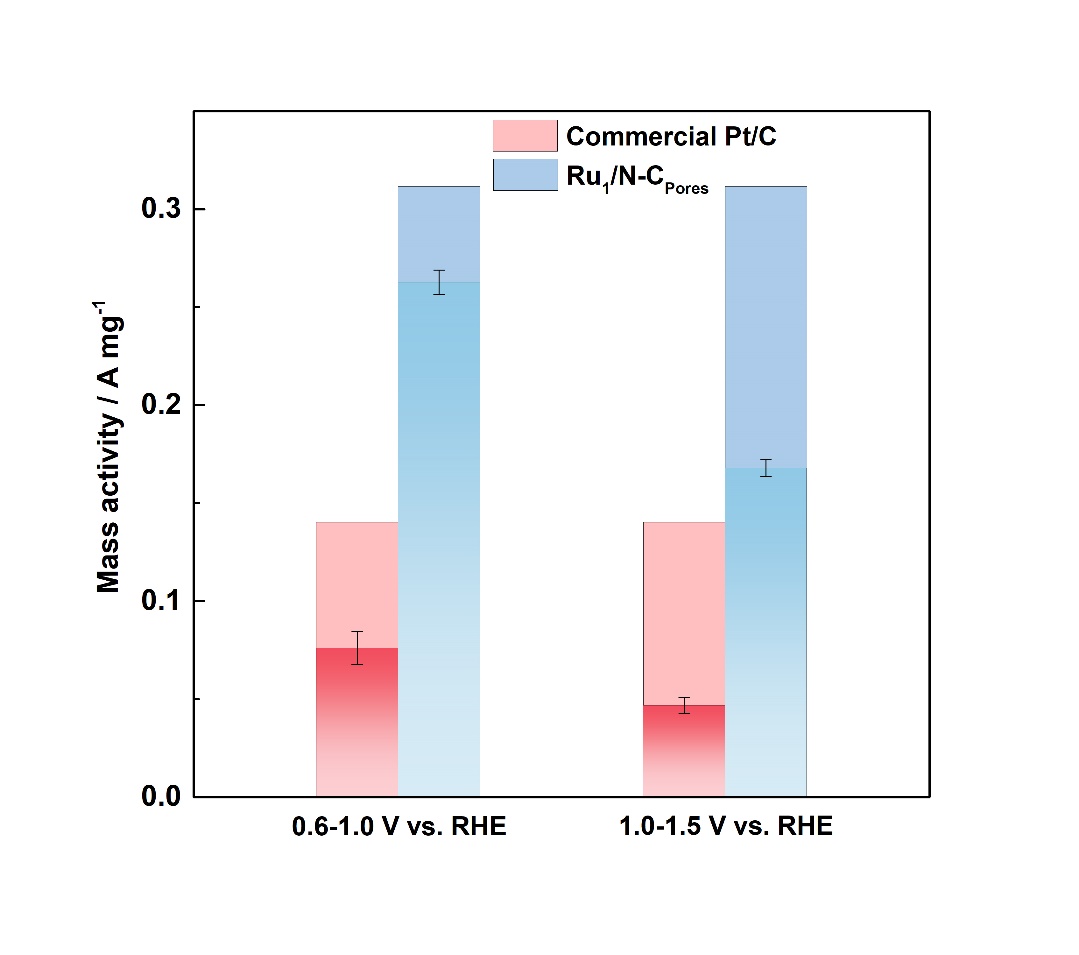


**Figure S15.** Mass activities of the catalysts at 0.9 V vs RHE before and after ADTs between 0.6-1.0 V vs RHE and 1.0-1.5 V vs RHE.


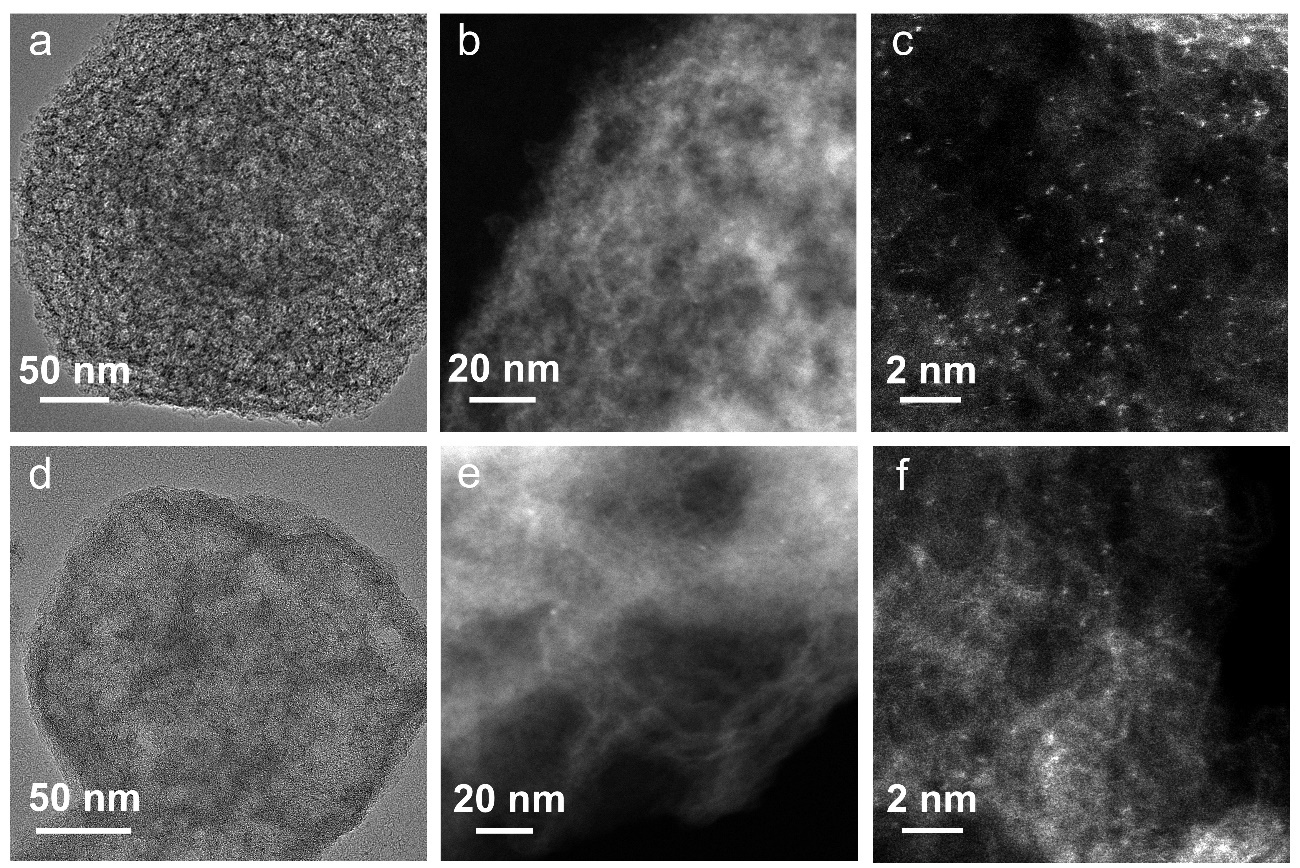


**Figure S16.** Morphology characterization of Ru_1_/N-C_Pores_ catalyst after ADTs between 0.6-1.0 V vs. RHE potential range: a) TEM image; b) HAADF-STEM image; c) Atomic-resolution HAADF-STEM image; Morphology characterization of Ru_1_/N-C_Pores_ catalyst after ADTs between 1.0-1.5 V vs. RHE potential range: d) TEM image; e) HAADF-STEM image; f) Atomic-resolution HAADF-STEM image.


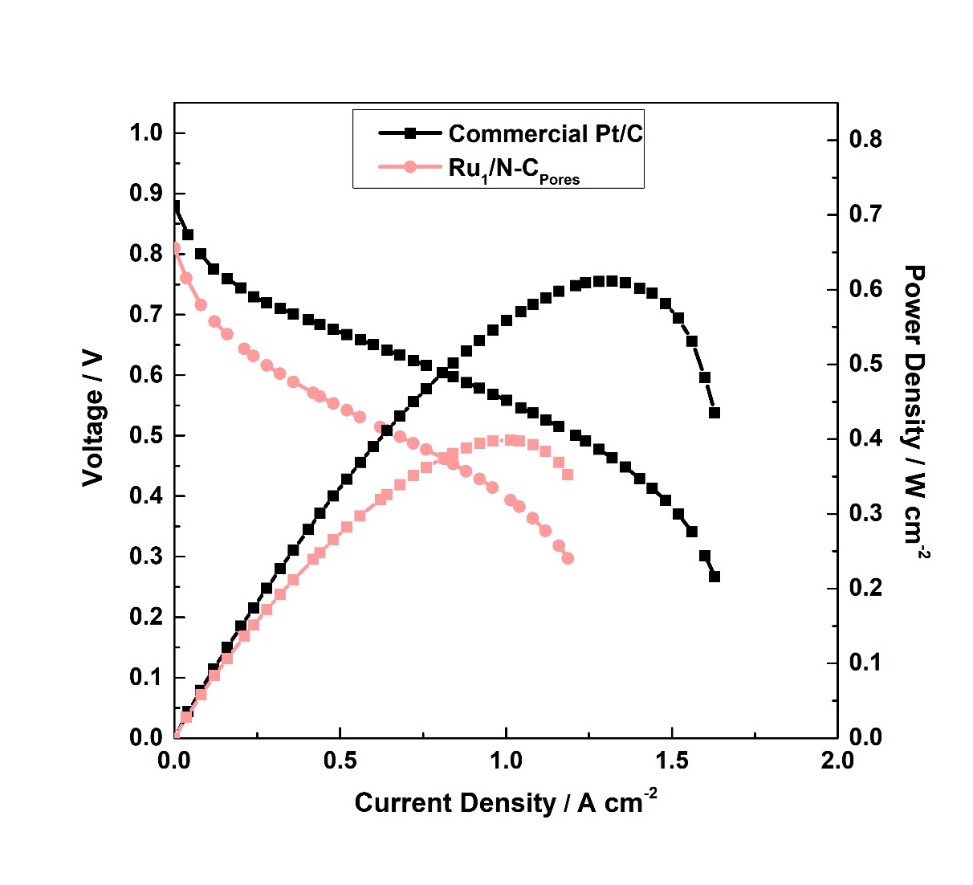


**Figure S17.** Polarization curves and power density of H_2_/air fuel cell employing different MEA cathode catalysts.


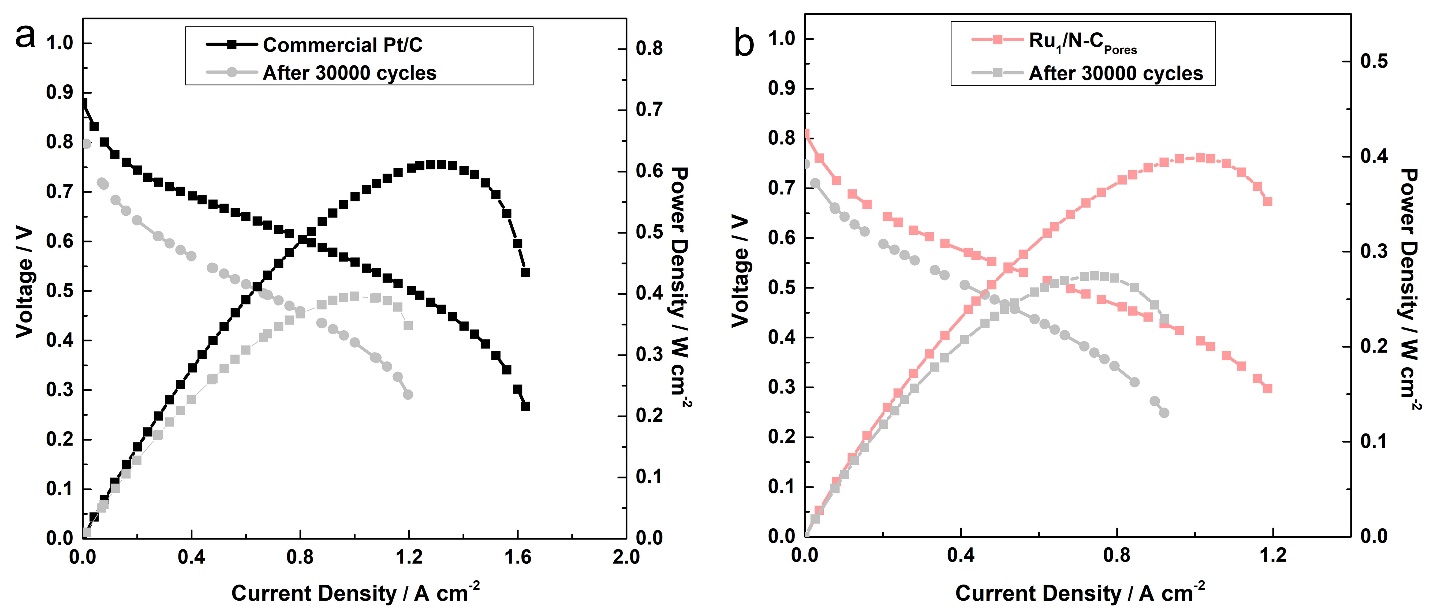


**Figure S18.** Polarization curves and power density of H_2_/air fuel cell employing different MEA cathode catalysts before and after ADTs: a) Commercial Pt/C; b) Ru_1_/N-C_Pores_.


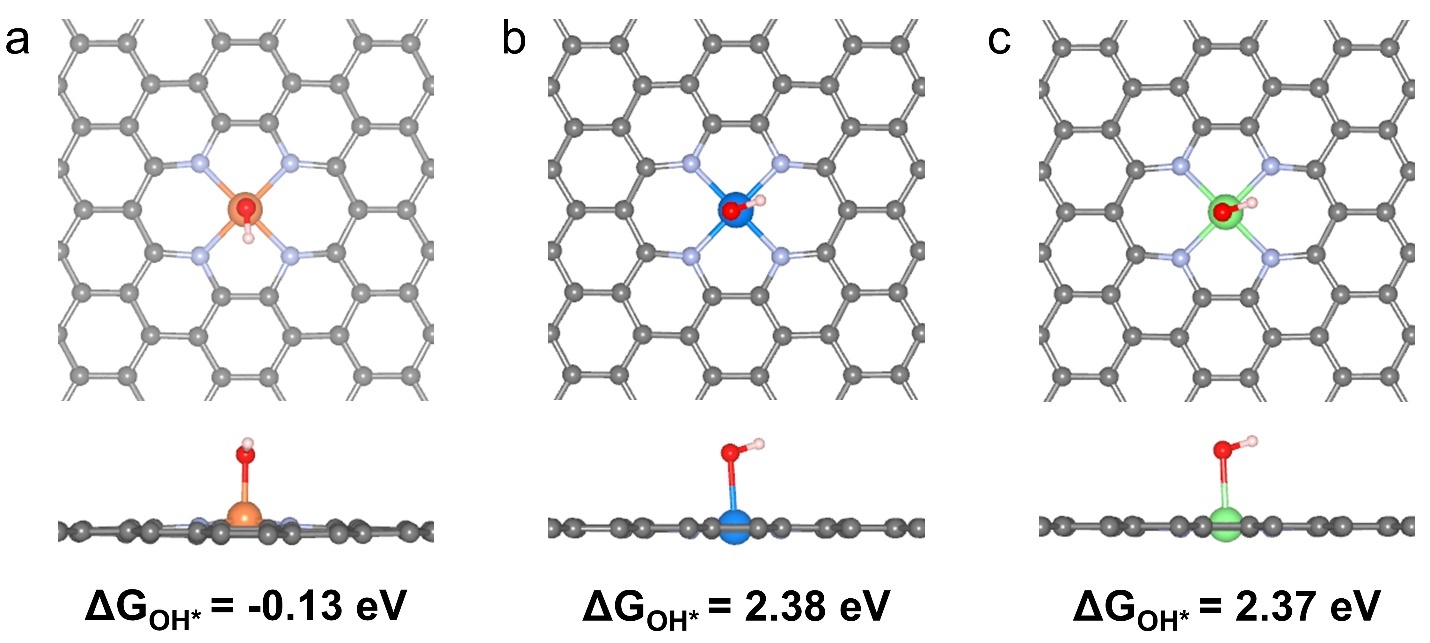


**Figure S19.** Top and side view of the adsorbed OH^*^ on different systems: a) RuN_4_; b) PtN_4_; c) PdN_4_.


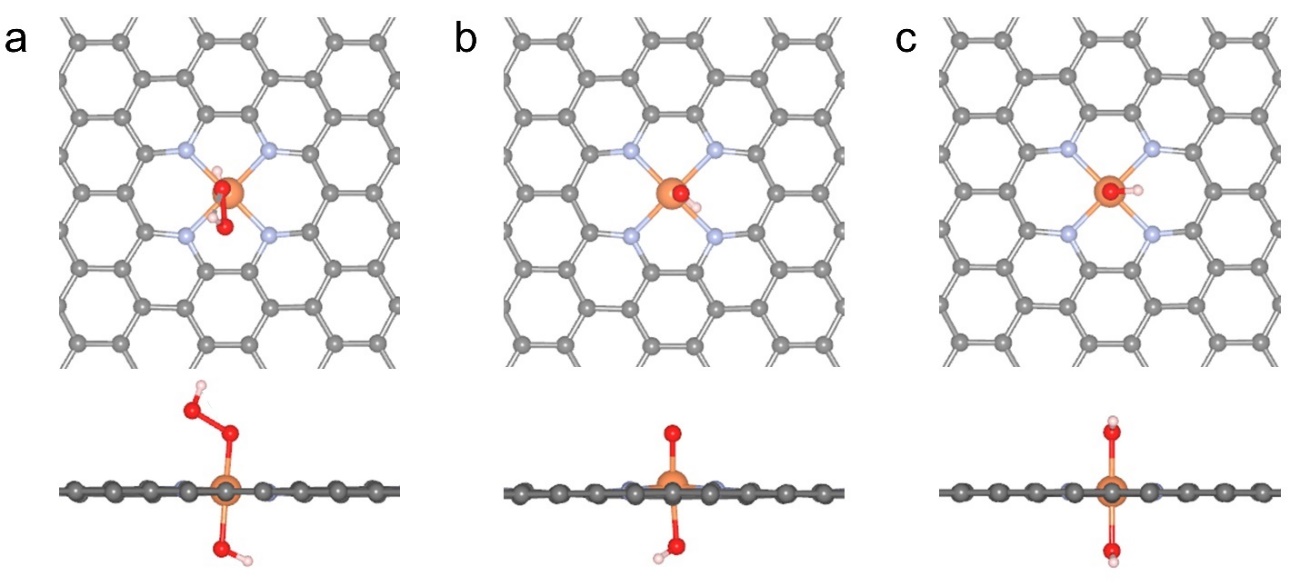


**Figure S20.** Top and side view of different ORR intermediates adsorbed on RuN_4_-OH: a) OOH^*^; b) O^*^; c) OH^*^.


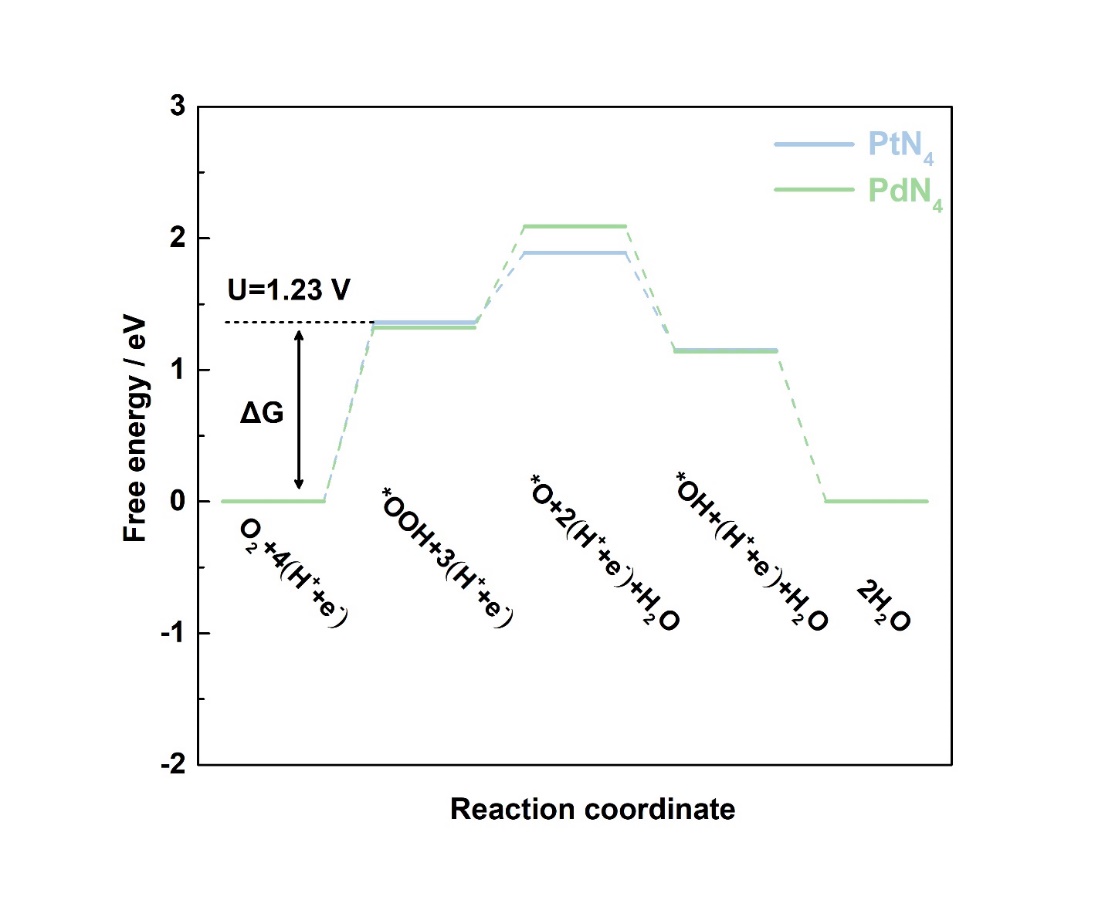


**Figure S21.** The calculated free energy plots of the ORR steps on PtN_4_ and PdN_4_ systems at the equilibrium potential of U=1.23 V vs. RHE.

**
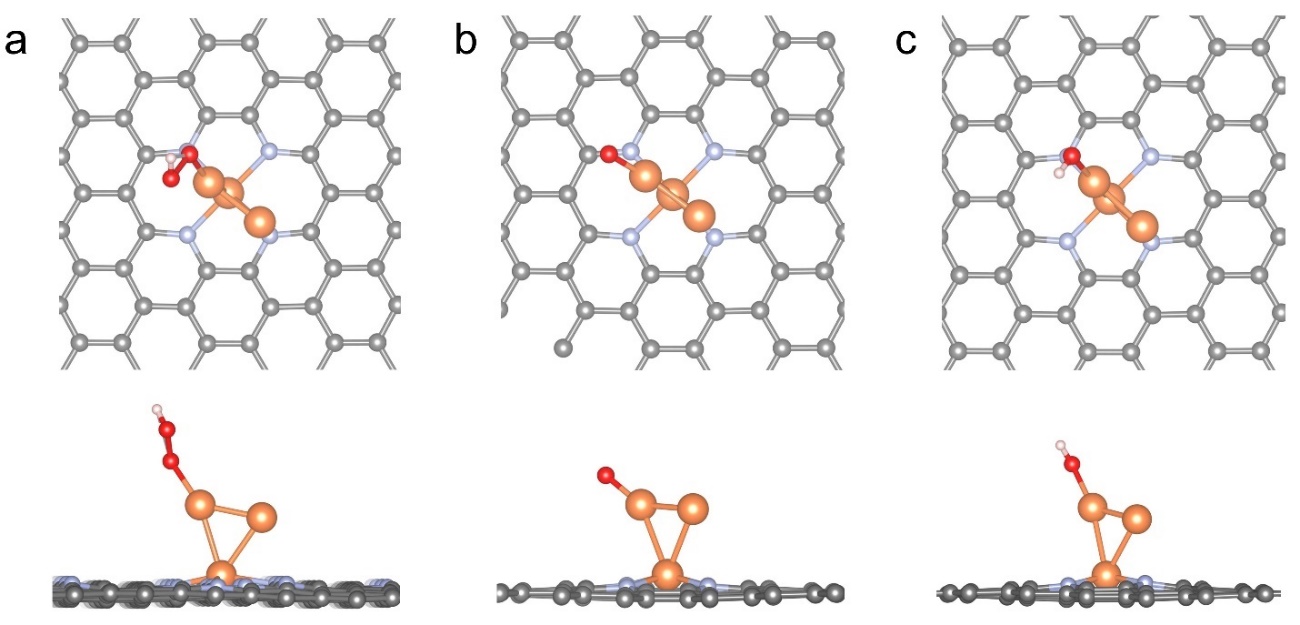
**

**Figure S22.** Top and side view of different ORR intermediates adsorbed on Ru_3_N_4_: a) OOH^*^; b) O^*^; c) OH^*^.

**Table S1.** Elemental analysis results of catalysts obtained from ICP-OES.

| Sample | | C (wt.%) | N (wt.%) | Zn (wt%) | PM (wt.%) |
| --- | --- | --- | --- | --- | --- |
| Ru_1_/N-C_Pores_ | As-prepared | 92.15 | 6.69 | 0.15 | 1.01 |
|  | After ADTs between 0.6-1.0 V vs RHE | 92.33 | 6.59 | 0.11 | 0.97 |
|  | After ADTs between 1.0-1.5 V vs RHE | 93.02 | 6.07 | 0.10 | 0.81 |
| Pt_1_/N-C_Pores_ | | 92.39 | 6.34 | 0.30 | 0.97 |
| Pd_1_/N-C_Pores_ | | 93.19 | 5.58 | 0.11 | 1.12 |
| Ru_x_/N-C | | 88.53 | 7.08 | 3.05 | 1.34 |
| Pt_x_/N-C | | 88.33 | 7.91 | 2.70 | 1.06 |
| Pd_x_/N-C | | 89.49 | 6.90 | 2.64 | 0.97 |

**Table S2.** Elemental analysis results of carbon supports obtained from ICP-OES.

| Sample | C (wt. %) | N (wt. %) | Zn (wt. %) |
| --- | --- | --- | --- |
| N-C | 90.22 | 7.01 | 2.77 |
| N-C_Pores_(1:1) | 92.70 | 6.07 | 1.23 |
| N-C_Pores_ | 92.25 | 6.86 | 0.89 |
| N-C_Pores_(1:4) | 90.55 | 6.89 | 2.56 |

**Table S3.** N content analysis results obtained from XPS.

| Sample | Pyridinic N  (%) | Pyrrolic N  (%) | Ru-N_x_  (%) | Graphitic N  (%) | Oxidized-N  (%) |
| --- | --- | --- | --- | --- | --- |
| N-C | 26.77 | 19.69 | - | 39.37 | 14.17 |
| N-C_Pores_(1:1) | 29.66 | 17.87 | - | 38.02 | 14.45 |
| N-C_Pores_ | 32.43 | 12.16 | - | 45.05 | 10.36 |
| N-C_Pores_(1:4) | 29.25 | 15.67 | - | 42.37 | 12.71 |
| Ru_1_/N-C_Pores_ | 25.55 | 9.49 | 18.61 | 36.50 | 9.85 |
| Ru_x_/N-C | 18.77 | 16.72 | 15.36 | 34.13 | 15.02 |

**Table S4.** BET results of carbon supports and catalysts.

| Sample | S_BET_  (m^2^ g^-1^) | S_external_  (m^2^ g^-1^) | V_pores_  (cm^3^ g^-1^) | V_micro_  (cm^3^ g^-1^) | Pore width  (nm) |
| --- | --- | --- | --- | --- | --- |
| N-C | 1147.98 | 55.95 | 0.63 | 0.45 | 2.19 |
| N-C_Pores_(1:1) | 1967.58 | 240.23 | 1.62 | 0.80 | 3.29 |
| N-C_Pores_ | 2103.99 | 233.86 | 1.74 | 0.86 | 3.30 |
| N-C_Pores_(1:4) | 1714.81 | 92.90 | 0.96 | 0.68 | 2.25 |
| Ru_1_/N-C_Pores_ | 2054.50 | 198.70 | 1.45 | 0.78 | 2.83 |
| Ru_x_/N-C | 1032.75 | 112.71 | 0.60 | 0.38 | 2.34 |

**Table S5.** EXAFS fitting parameters at the Ru K–edge for Ru foil, RuO_2_, Ru_1_/N-C_Pores_ and Ru_x_/N-C.

| Sample | Shell | CN | R(Å) | σ^2^(Å^2^) | ΔE_0_(eV) | R-factor |
| --- | --- | --- | --- | --- | --- | --- |
| Ru foil | Ru-Ru | 12* | 2.67±0.01 | 0.0033 | 4.6 | 0.0037 |
| RuO_2_ | Ru-O | 6.1±0.4 | 1.95±0.01 | 0.0023 | 10.5 | 0.0183 |
|  | Ru-Ru | 8.7±2.0 | 3.13±0.01 | 0.0109 | 9.3 |  |
|  | Ru-Ru | 7.4±0.6 | 3.56±0.01 | 0.0028 | 10.9 |  |
| Ru_1_/N-C_Pores_ | Ru-N(O) | 4.0±0.3 | 2.00±0.01 | 0.0046 | 10.4 | 0.0134 |
| Ru_x_/N-C | Ru-N(O) | 3.8±0.6 | 2.02±0.01 | 0.0069 | 12.4 | 0.0176 |
|  | Ru-Ru | 2.0±0.6 | 2.67±0.01 | 0.0042 | 6.3 |  |

**Table S6.** Catalytic performance comparison of Ru_1_/N-C_Pores_ catalyst with recently reported single atom catalysts in acid electrolyte.

| Catalyst | Catalyst loading  (mg·cm^-2^) | *E*_onset_  (V) | *E*_1/2_  (V) | TOF  (e^-^ site^-1^ s^-1^) | Reference |
| --- | --- | --- | --- | --- | --- |
| Ru_1_/N-C_Pores_ | 0.30 | 0.980 | 0.835 | 6.19 | This work |
| Pt_1_-N/BP | 0.39 | - | 0.76 | - | [10] |
| Ir-SAC | 0.40 | 0.970 | 0.864 | 24.3 | [11] |
| Ir_1_-N-HsGDY | 0.4 | 0.90 | 0.77 |  | [12] |
| Ru-SSC | 0.60 | 0.920 | 0.824 | 4.99 | [13] |
| Ru-N/G-750 | 0.32 | 0.89 | 0.75 | 1 | [14] |
| Ru-SA/Ti_3_C_2_T*_x_* | 0.12 | 0.92 | 0.8 |  | [15] |
| TPI@Z8(SiO2)-650-C | 0.40 | - | 0.823 | 1.60 | [16] |
| Fe/NC-NaCl-1 | 1.2 | - | 0.832 | 0.83 | [17] |
| Fe-N-C-3HT-2AL | 0.8 | 0.94 | 0.84 | 1.4 | [18] |
| Fe_SA_-N-C | 0.28 | 0.95 | 0.80 |  | [19] |

**Table S7.** Catalytic performance of commercial Pt/C, Ru_1_/N-C_Pores_ and Ru_x_/N-C catalysts (Data from six repeated tests).

| Sample | *E*_1/2_  (V) | *j*_k,mass_ at 0.8 V vs RHE  (A·mg_Pt_^-1^) | | *j*_k,mass_ at 0.9 V vs RHE  (A·mg_Pt_^-1^) | |
| --- | --- | --- | --- | --- | --- |
| Commercial Pt/C | 0.856 | 1.13 | 1.09±0.16 | 0.14 | 0.14±0.01 |
|  | 0.862 | 1.00 |  | 0.15 |  |
|  | 0.857 | 1.25 |  | 0.14 |  |
|  | 0.859 | 1.31 |  | 0.13 |  |
|  | 0.854 | 0.92 |  | 0.15 |  |
|  | 0.860 | 0.95 |  | 0.15 |  |
| Ru_1_/N-C_Pores_ | 0.835 | 5.12 | 5.83±0.61 | 0.32 | 0.31±0.02 |
|  | 0.837 | 5.44 |  | 0.33 |  |
|  | 0.835 | 5.49 |  | 0.33 |  |
|  | 0.837 | 6.83 |  | 0.28 |  |
|  | 0.830 | 6.17 |  | 0.29 |  |
|  | 0.833 | 5.91 |  | 0.33 |  |
| Ru_x_/N-C | 0.725 | 0.35 | 0.36±0.04 | 0.05 | 0.05±0.01 |
|  | 0.731 | 0.43 |  | 0.05 |  |
|  | 0.727 | 0.36 |  | 0.05 |  |
|  | 0.727 | 0.33 |  | 0.05 |  |
|  | 0.730 | 0.36 |  | 0.05 |  |
|  | 0.724 | 0.33 |  | 0.04 |  |

**Table S8.** Catalytic performance results of commercial Pt/C and Ru_1_/N-C_Pores_ after ADTs between 0.6-1.0 V vs RHE (Data from three repeated tests).

| Sample | *E*_1/2_  (V) | *j*_k,mass_ at 0.9 V vs RHE  (A·mg_Pt_^-1^) | |
| --- | --- | --- | --- |
| Commercial Pt/C | 0.832 | 0.07 | 0.08±0.01 |
|  | 0.837 | 0.09 |  |
|  | 0.830 | 0.07 |  |
| Ru_1_/N-C_Pores_ | 0.819 | 0.26 | 0.26±0.01 |
|  | 0.823 | 0.26 |  |
|  | 0.820 | 0.27 |  |

**Table S9.** Catalytic performance results of commercial Pt/C and Ru_1_/N-C_Pores_ after ADTs between 1.0-1.5 V vs. RHE (Data from three repeated tests).

| Sample | *E*_1/2_  (V) | *j*_k,mass_ at 0.9 V vs RHE  (A·mg_Pt_^-1^) | |
| --- | --- | --- | --- |
| Commercial Pt/C | 0.819 | 0.05 | 0.05±0.01 |
|  | 0.823 | 0.04 |  |
|  | 0.820 | 0.05 |  |
| Ru_1_/N-C_Pores_ | 0.808 | 0.17 | 0.17±0.01 |
|  | 0.802 | 0.16 |  |
|  | 0.805 | 0.17 |  |

**Table S10.** Calculated Gibbs free energies for ORR intermediates on RuN_4_, PtN_4_, PdN_4_, RuN_4_-OH and Ru_3_N_4_ systems at U=0 and 1.23 V vs. RHE.

| U (V) | Model | $\text{∆}\text{G}_{\mathrm{OOH}^{*}} ($eV) | $\text{∆}\text{G}_{O^{*}}$(eV) | $\text{∆}\text{G}_{\mathrm{OH}^{*}} ($eV) |
| --- | --- | --- | --- | --- |
| 0 | RuN_4_ | 2.73 | 0.53 | -0.13 |
|  | PtN_4_ | 5.05 | 4.35 | 2.38 |
|  | PdN_4_ | 5.01 | 4.55 | 2.37 |
|  | RuN_4_-OH | 3.21 | 1.35 | 0.59 |
|  | Ru_3_N_4_ | 2.74 | -0.40 | -0.53 |
| 1.23 | RuN_4_ | -0.96 | -1.93 | -1.26 |
|  | PtN_4_ | 1.36 | 1.89 | 1.15 |
|  | PdN_4_ | 1.32 | 2.09 | 1.14 |
|  | RuN_4_-OH | -0.48 | -1.11 | -0.64 |
|  | Ru_3_N_4_ | -0.95 | -2.86 | -1.76 |

References

[1] G. Kresse, J. Furthmüller, *Phys. Rev. B* **1996**, 54, 11169.

[2] G. Kresse, J. Furthmüller, *Comp. Mater. Sci.* **1996**, 6, 15-50.

[3] J. P. Perdew, K. Burke, M. Ernzerhof, *Phys. Rev. Lett.* **1996**, 77, 3865.

[4] P. E. Blöchl, *Phys. Rev. B* **1994**, 50, 17953.

[5] S. Grimme, J. Antony, S. Ehrlich, H. Krieg, *J. Chem. Phys.* **2010**, 132, 154104.

[6] H. J. Monkhorst, J. D. Pack, *Phys. Rev. B* **1976**, 13, 5188.

[7] K. Momma, F. Izumi, *J. Appl. Crystallogr.* **2011**, 44, 1272-1276.

[8] J. K. Nørskov, J. Rossmeisl, A. Logadottir, L. Lindqvist, J. R. Kitchin, T. Bligaard, H. Jonsson, *J. Phys. Chem. B* **2004**, 108, 17886-17892.

[9] M. Bajdich, M. Garcia-Mota, A. Vojvodic, J. K. Norskov, A. T. Bell, *J. Am. Chem. Soc.* **2013**, 135, 13521-13530.

[10] J. Liu, M. Jiao, L. Lu, H. M. Barkholtz, Y. Li, Y. Wang, L. Jiang, Z. Wu, D. J. Liu, L. Zhuang, C. Ma, J. Zeng, B. Zhang, D. Su, P. Song, W. Xing, W. Xu, Y. Wang, Z. Jiang, G. Sun, *Nat. Commun.* **2017**, 8, 15938.

[11] M. Xiao, J. Zhu, G. Li, N. Li, S. Li, Z. P. Cano, L. Ma, P. Cui, P. Xu, G. Jiang, *Angew. Chem. Int. Ed.* **2019**, 58, 9640-9645.

[12] Q. Lv, M. Li, X. Li, X. Yan, Z. Hou, C. Huang, *J Energy Chem.* **2024**, 1, 144-151.

[13] M. Xiao, L. Gao, Y. Wang, X. Wang, J. Zhu, Z. Jin, C. Liu, H. Chen, G. Li, J. Ge, *J. Am. Chem. Soc.* **2019**, 141, 19800-19806.

[14] C. Zhang, J. Sha, H. Fei, M. Liu, S. Yazdi, J. Zhang, Q. Zhong, X. Zou, N. Zhao, H. Yu, *ACS Nano* **2017**, 11, 6930-6941.

[15] X. Peng, S. Zhao, Y. Mi, L. Han, X. Liu, D. Qi, J. Sun, Y. Liu, H. Bao, L. Zhuo, H, Xin, J. Luo, X. Sun, *Small* **2020**, 16, 2002888.

[16] X. Wan, X. Liu, Y. Li, R. Yu, L. Zheng, W. Yan, H. Wang, M. Xu and J. Shui, *Nat. Catal.* **2019**, *2*, 259

[17] Q. Wang, Y. Yang, F. Sun, G. Chen, J. Wang, L. Peng, W. T. Chen, L. Shang, J. Zhao, D. Sun‐Waterhouse, *Adv. Energy Mater.* **2021**, 11, 2100219.

[18] N. R. Sahraie, U. I. Kramm, J. Steinberg, Y. Zhang, A. Thomas, T. Reier, J. P. Paraknowitsch, P. Strasser, *Nat. Commun.* **2015**, 6, 8618.

[19] L. Jiao, R. Zhang, G. Wan, W. Yang, X. Wan, H. Zhou, J. Shui, S. H. Yu, H. L. Jiang, *Nat. Commun.* **2020**, 11, 2831.
